# Spectral data for cholestane glycosides from the bulbs of *Ornithogalum saundersiae* Baker

**DOI:** 10.1016/j.dib.2019.104391

**Published:** 2019-08-12

**Authors:** Qing-Wei Chen, Xu Zhang, Ting Gong, Wan Gao, Shuai Yuan, Pei-Cheng Zhang, Jian-Qiang Kong

**Affiliations:** Institute of Materia Medica, Chinese Academy of Medical Sciences & Peking Union Medical College (State Key Laboratory of Bioactive Substance and Function of Natural Medicines & NHC Key Laboratory of Biosynthesis of Natural Products), Beijing, 100050, China

**Keywords:** *Ornithogalum saundersiae* Baker, Asparagaceae, Cholestane glycosides, Acylated steroidal glycoside

## Abstract

Herein, the spectral data, including nuclear magnetic resonance spectroscopy (NMR) and mass spectral data, and gas chromatography data of eight cholestane glycosides from *Ornithogalum saundersiae* Baker (Asparagaceae) bulbs are described. The data are linked with the article entitled “Structure and bioactivity of cholestane glycosides from the bulbs of *Ornithogalum saundersiae* Baker” (Chen et al., 2019).

**Table:** Specifications Table

Subject	*Chemistry*
Specific subject area	*Natural products research*
Type of data	*Figures*
How data were acquired	*High-resolution electrospray ionization mass spectrometry (HRESIMS), NMR, Gas chromatography (GC)*
Data format	*Raw, filtered and analyzed*
Parameters for data collection	*The purified isolates were subjected to HRESIMS analysis. Cholestane glycosides were dissolved in C5D5N prior to NMR analysis. The acidified compounds must be derivatized before GC analysis.*
Description of data collection	*HRESIMS was performed on an Agilent 6520 HPLC-Q-TOF. NMR data were recorded with Bruker AV-Ⅲ-500 spectrometer or a Bruker-*600 NMR *spectrometer. GC analysis were conducted in Agilent 7890 system.*
Data source location	*Institute of Materia Medica, Chinese Academy of Medical Sciences & Peking Union Medical College* *Beijing* *P. R. China*
Data accessibility	*Data is with this article*
Related research article	*Qing-Wei Chen, Xu Zhang, Ting Gong, Wan Gao, Shuai Yuan, Pei-Cheng Zhang, Jian-Qiang Kong* *Structure and bioactivity of cholestane glycosides from the bulbs of Ornithogalum saundersiae Baker* *Phytochemistry, 2019, 164:206-214*

**Table:** 

**Value of the data** • Spectral data of cholestane glycosides are useful for elucidating their chemical structures. • The presented data benefit the chemical researchers, especially those working on the structural identification of steroidal glycosides. • The presented data can provide references for the structural characterization of related cholestane glycosides in other species. • The method described in this article can provide references for the isolation of related compounds.

## 1 Data

Eight new cholestane glycosides were isolated from the bulbs of *Ornithogalum saundersiae* Baker [1]. Their spectral data, including NMR and mass spectral data, and gas chromatography data were presented in this article. See Fig. 1, Fig. 2, Fig. 3, Fig. 4, Fig. 5, Fig. 6, Fig. 7, Fig. 8, Fig. 9, Fig. 10, Fig. 11, Fig. 12, Fig. 13, Fig. 14, Fig. 15, Fig. 16, Fig. 17, Fig. 18, Fig. 19, Fig. 20, Fig. 21, Fig. 22, Fig. 23, Fig. 24, Fig. 25, Fig. 26, Fig. 27, Fig. 28, Fig. 29, Fig. 30, Fig. 31, Fig. 32, Fig. 33, Fig. 34, Fig. 35, Fig. 36, Fig. 37, Fig. 38, Fig. 39, Fig. 40, Fig. 41, Fig. 42, Fig. 43, Fig. 44, Fig. 45, Fig. 46, Fig. 47, Fig. 48, Fig. 49, Fig. 50, Fig. 51, Fig. 52, Fig. 53, Fig. 54, Fig. 55, Fig. 56, Fig. 57, Fig. 58, Fig. 59, Fig. 60, Fig. 61, Fig. 62, Fig. 63, Fig. 64, Fig. 65, Fig. 66 with this article.

**Fig. 1:**
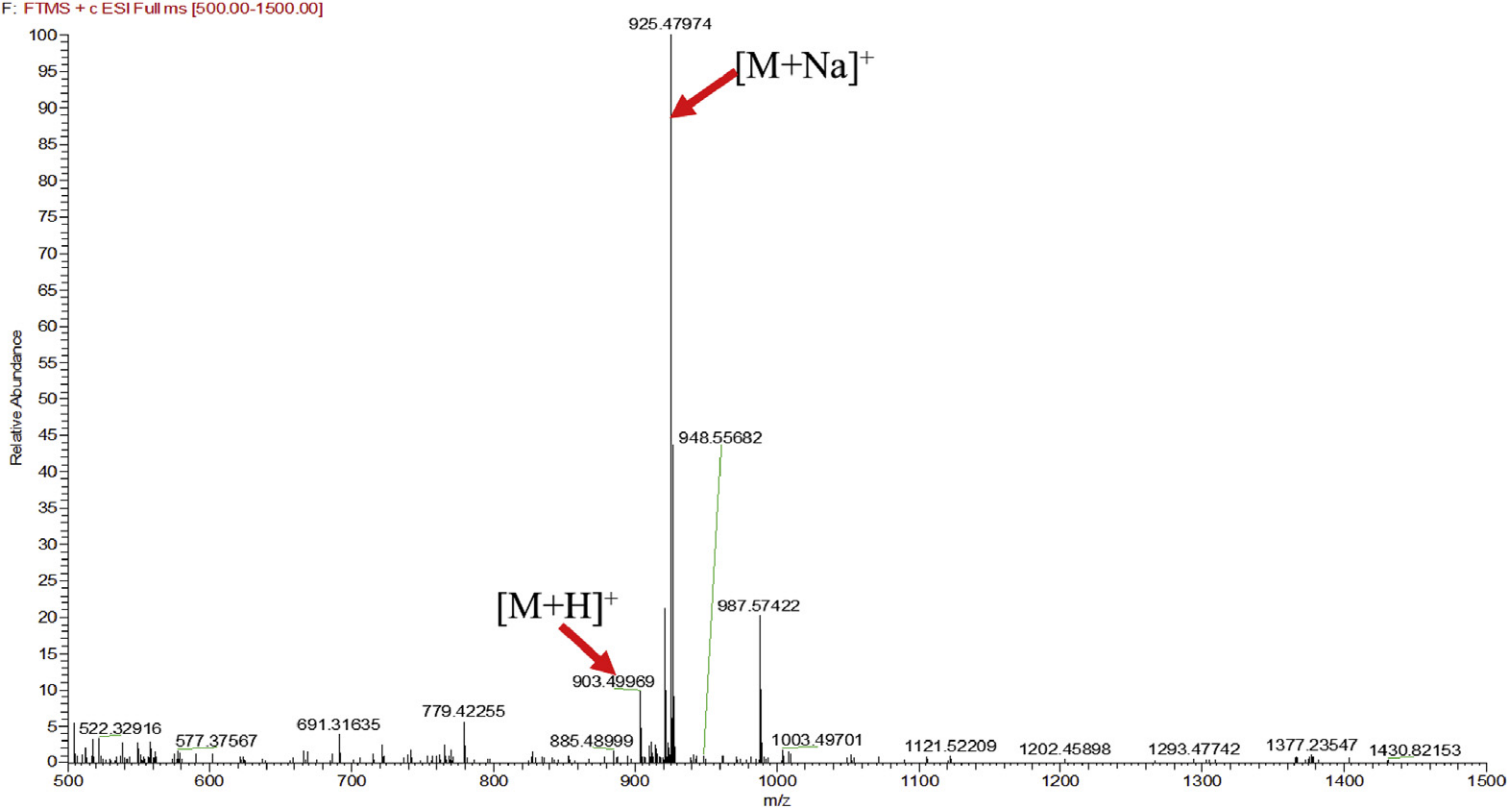
HRESIMS spectra of 1.

**Fig. 2:**
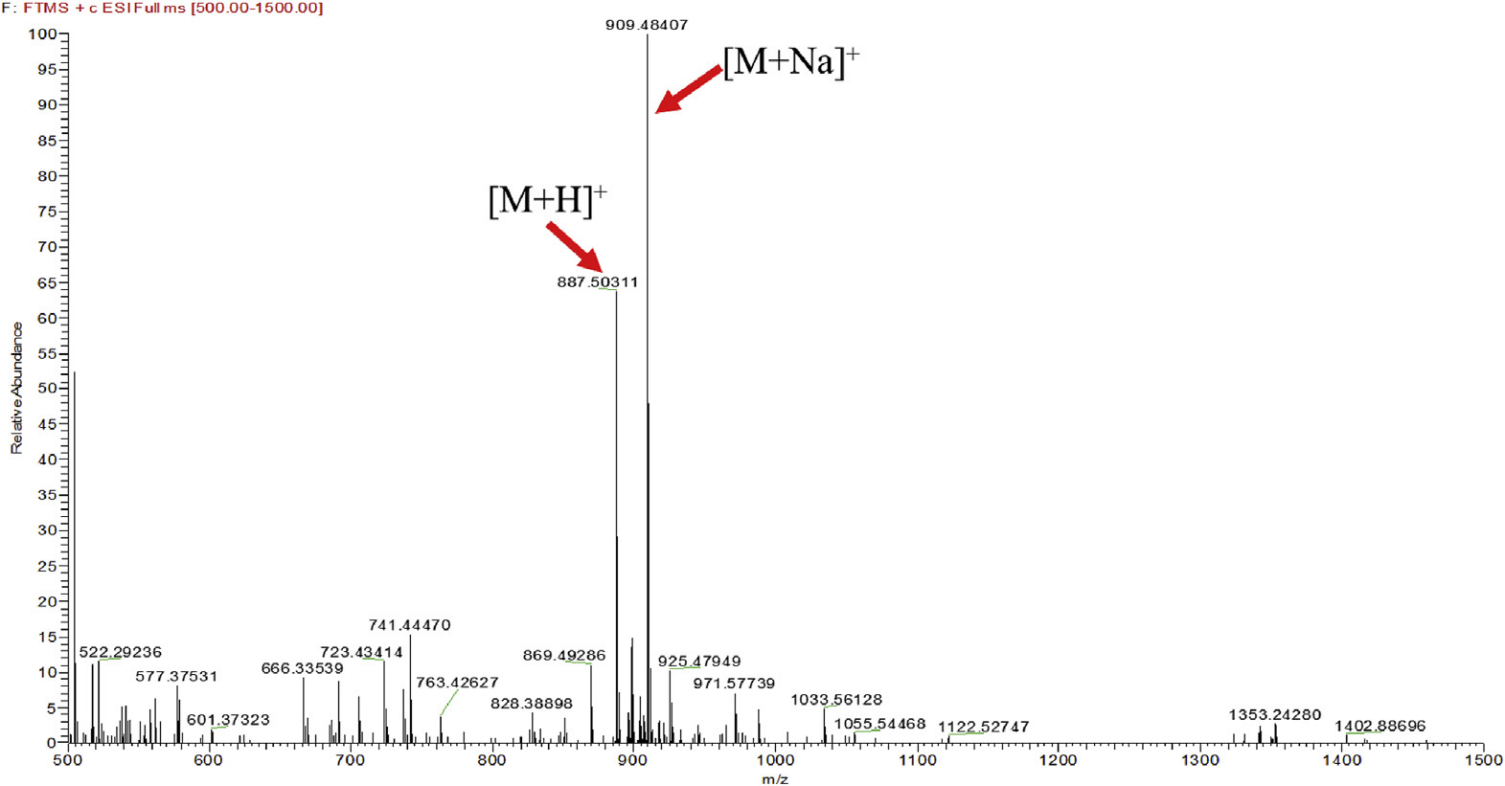
HRESIMS spectra of 2.

**Fig. 3:**
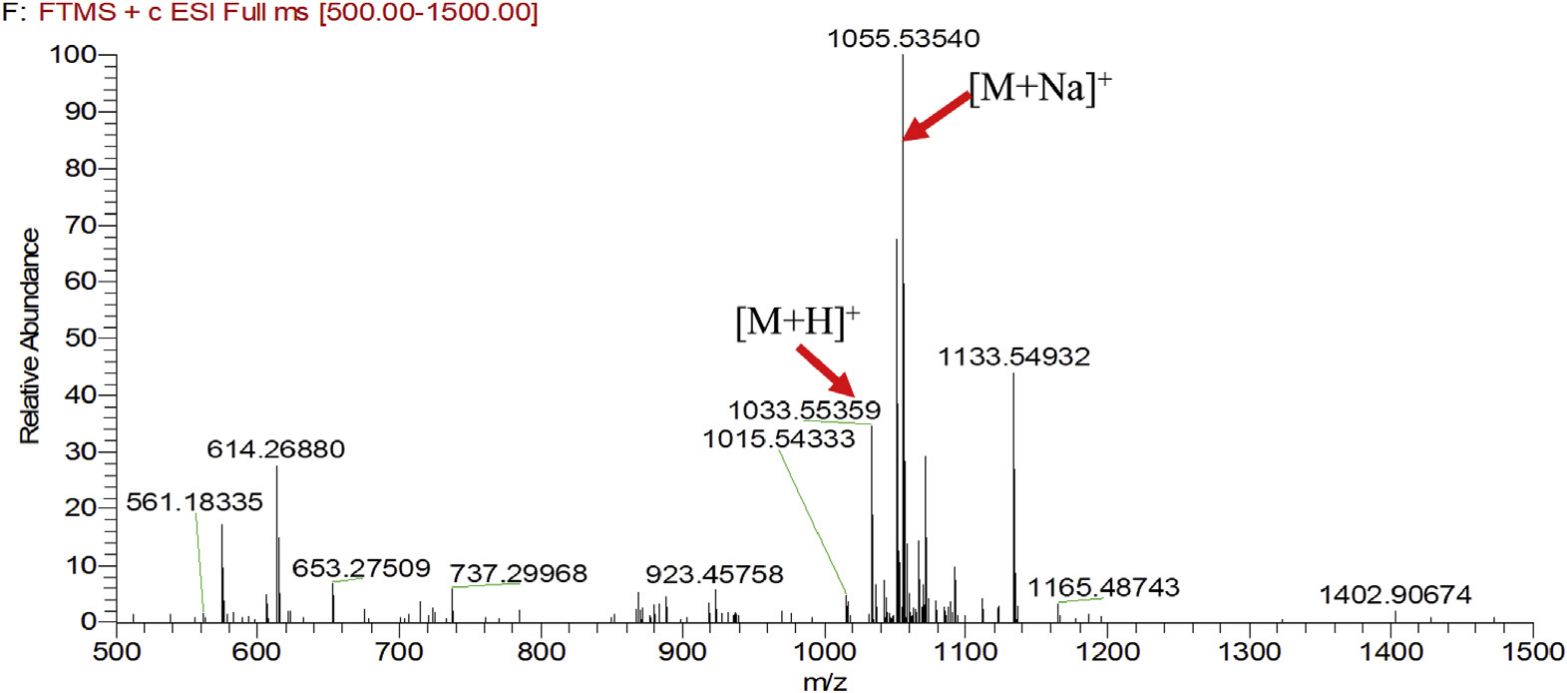
HRESIMS spectra of 3.

**Fig. 4:**
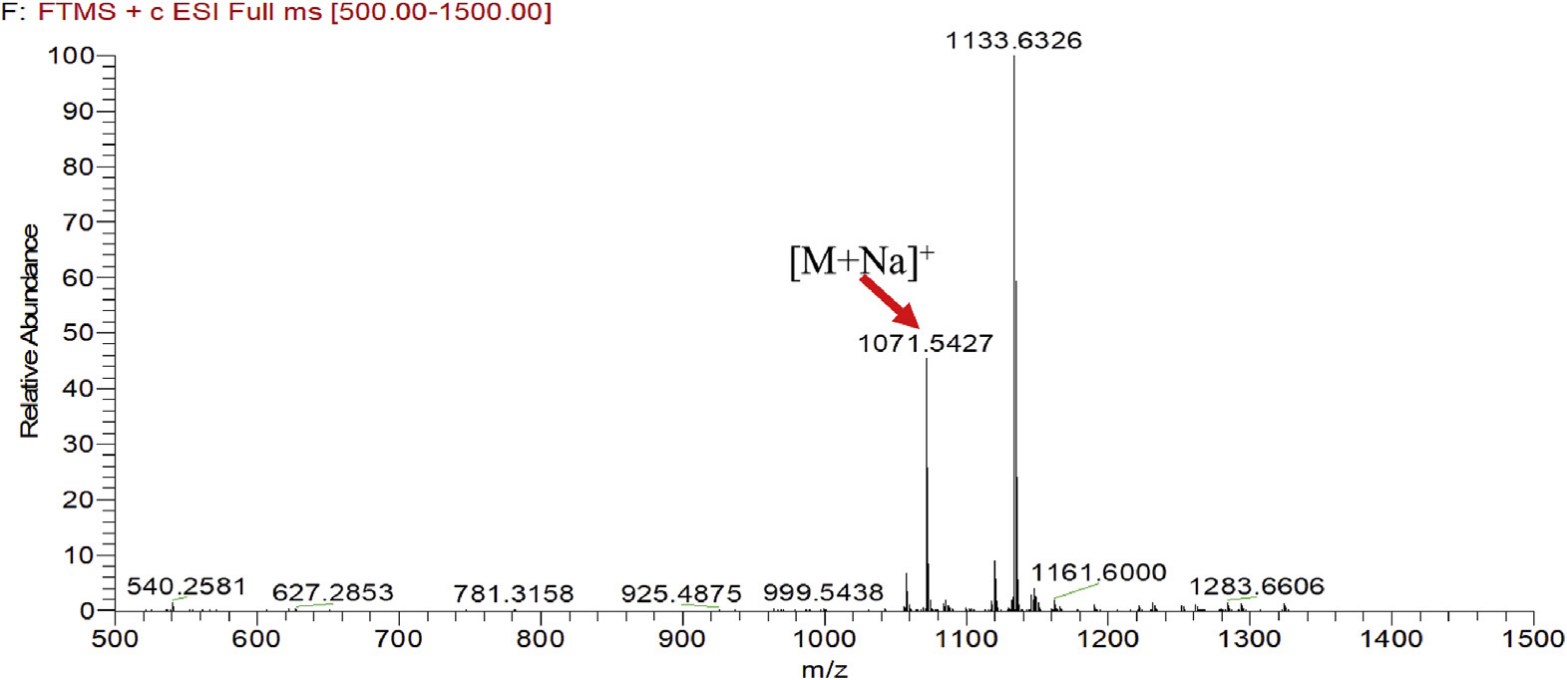
HRESIMS spectra of 4.

**Fig. 5:**
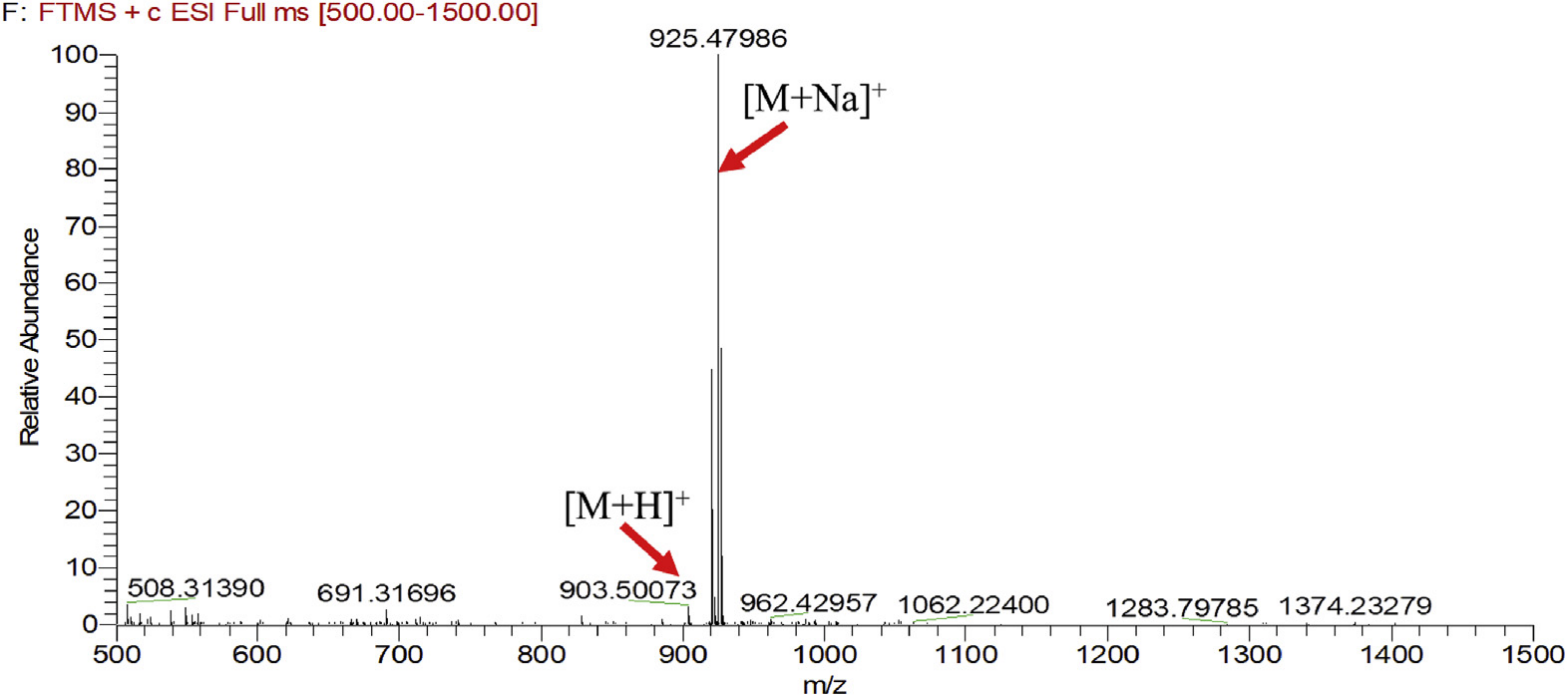
HRESIMS spectra of 5.

**Fig. 6:**
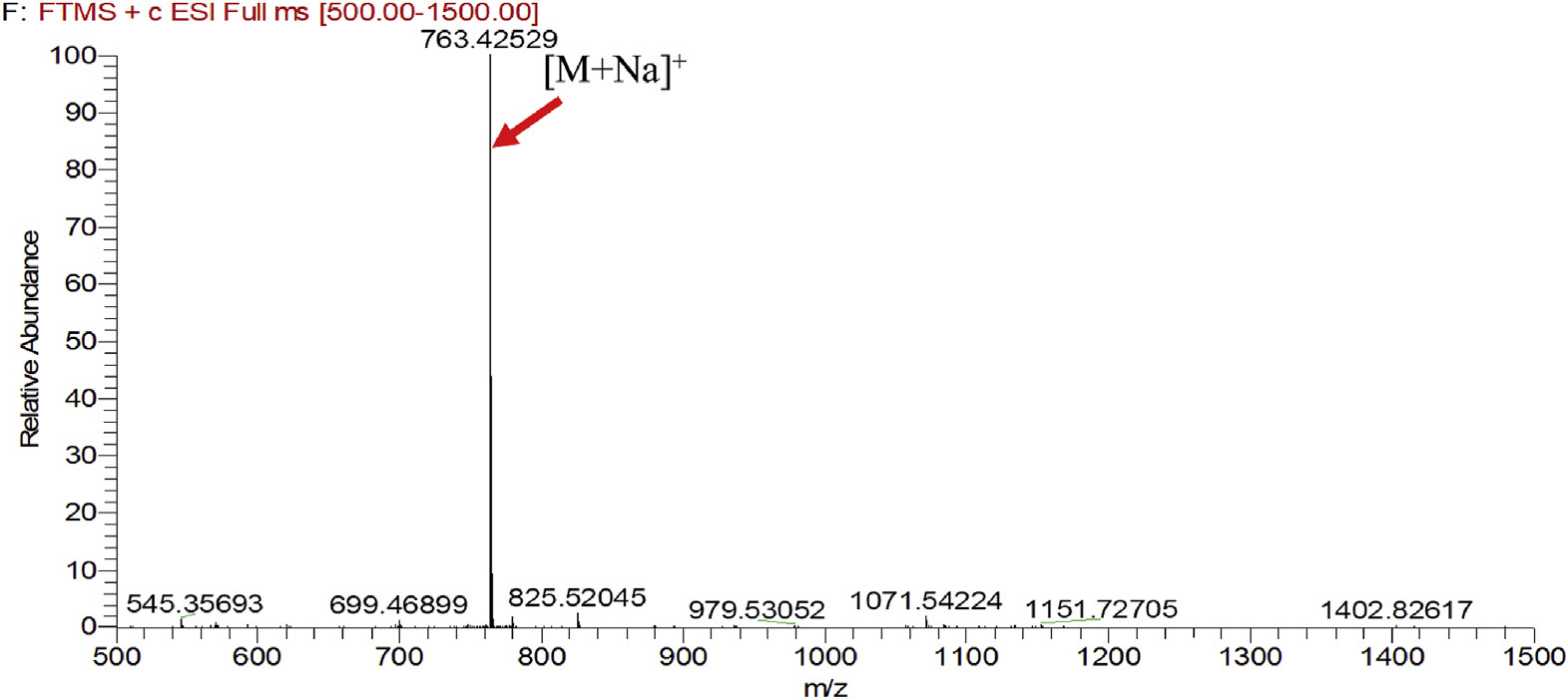
HRESIMS spectra of 6.

**Fig. 7:**
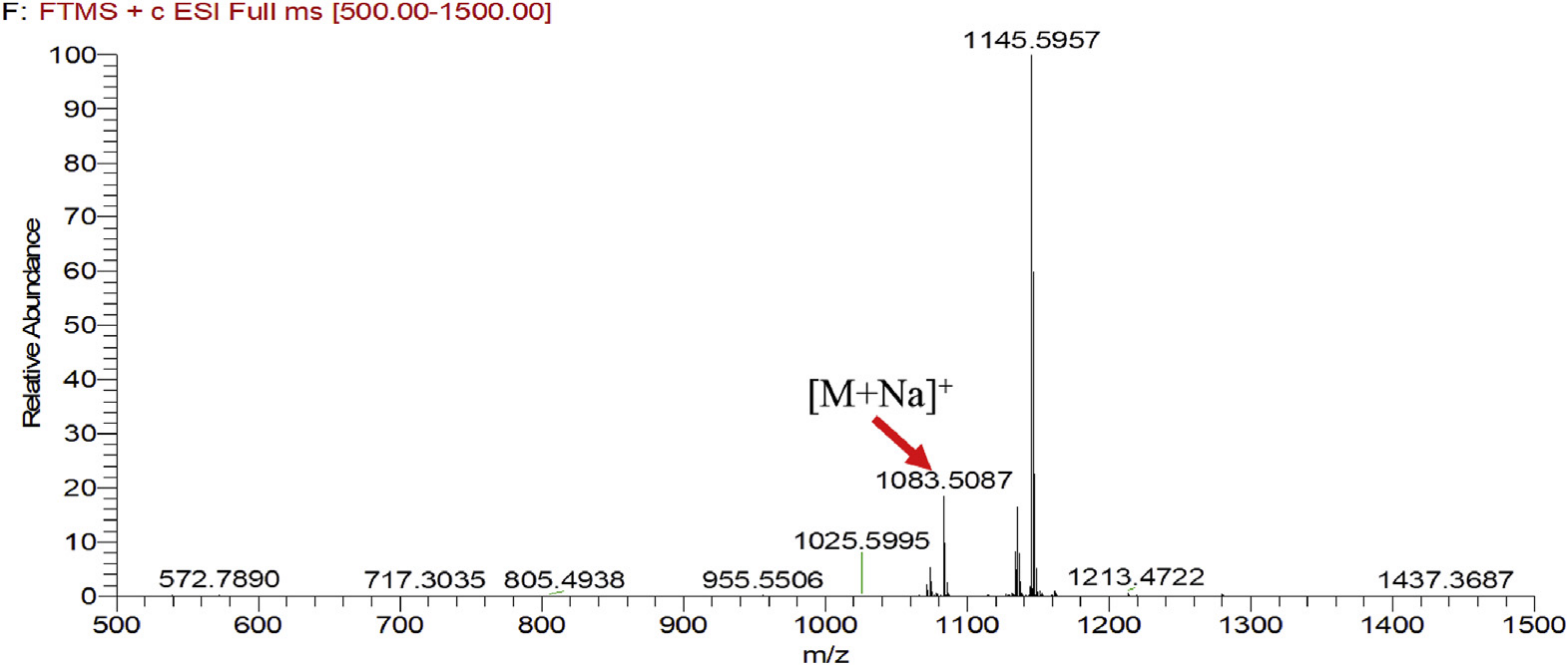
HRESIMS spectra of 7.

**Fig. 8:**
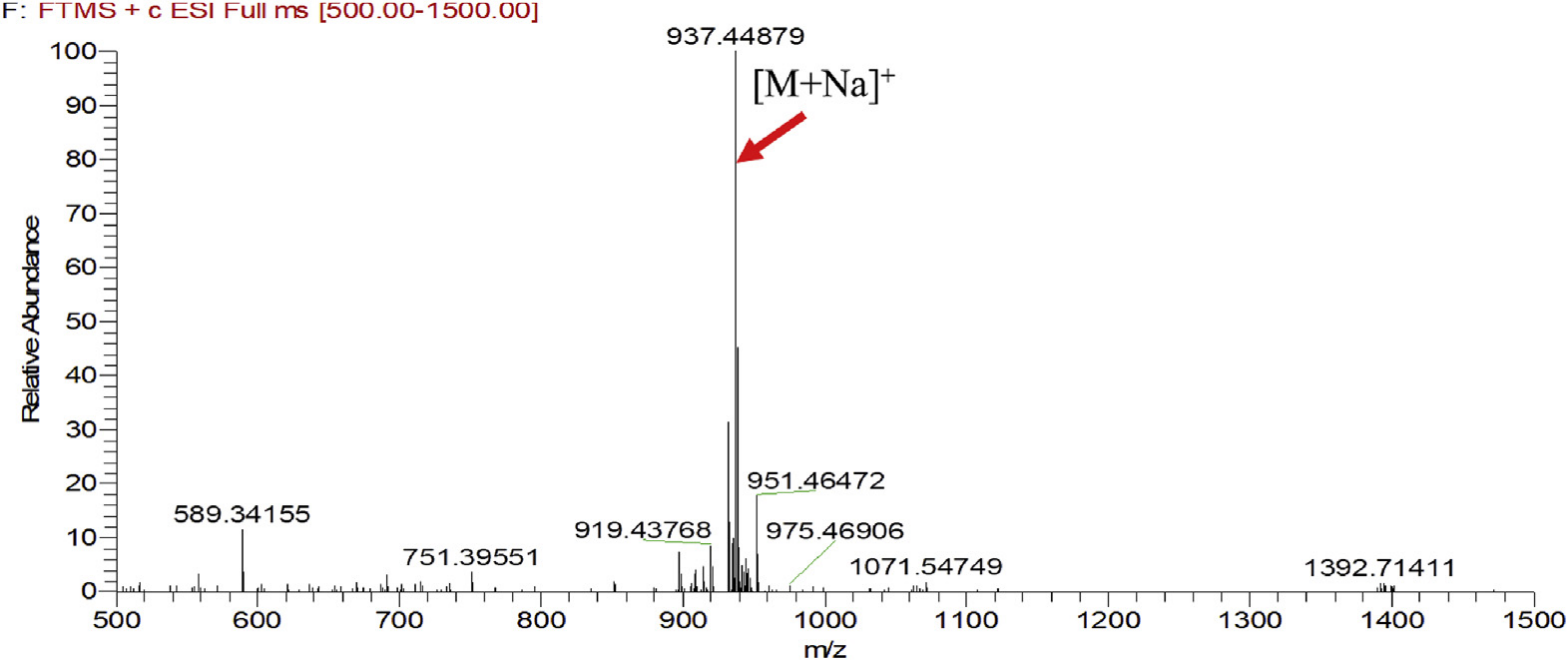
HRESIMS spectra of 8.

**Fig. 9:**
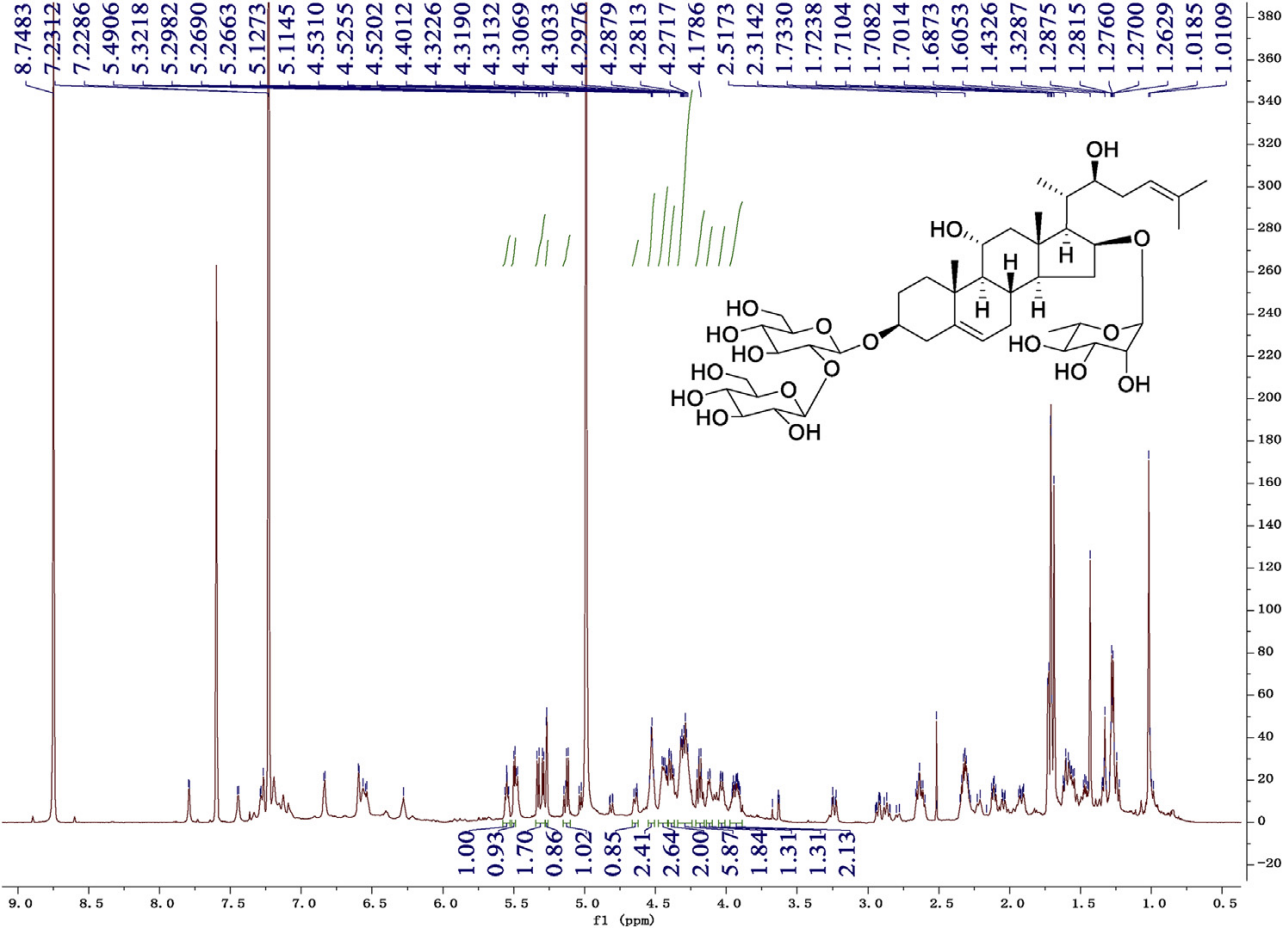
^1^H-NMR (600 MHz) spectrum in pyridine-*d*_5_ of 1.

**Fig. 10:**
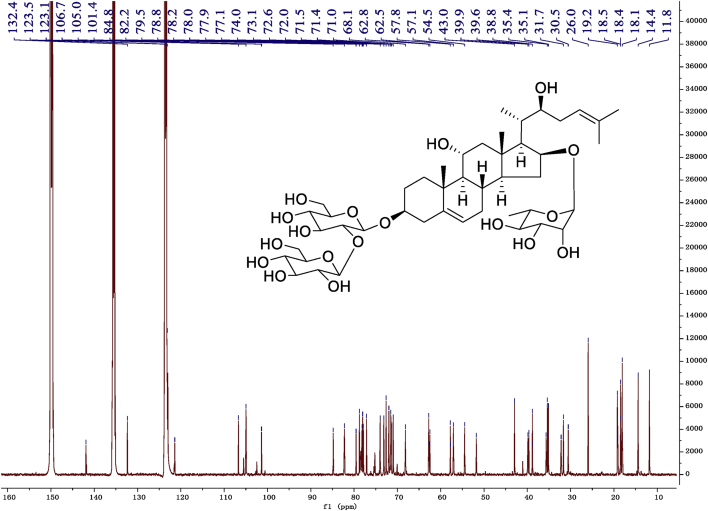
^13^C -NMR (150 MHz) spectrum in pyridine-*d*_5_ of 1.

**Fig. 11:**
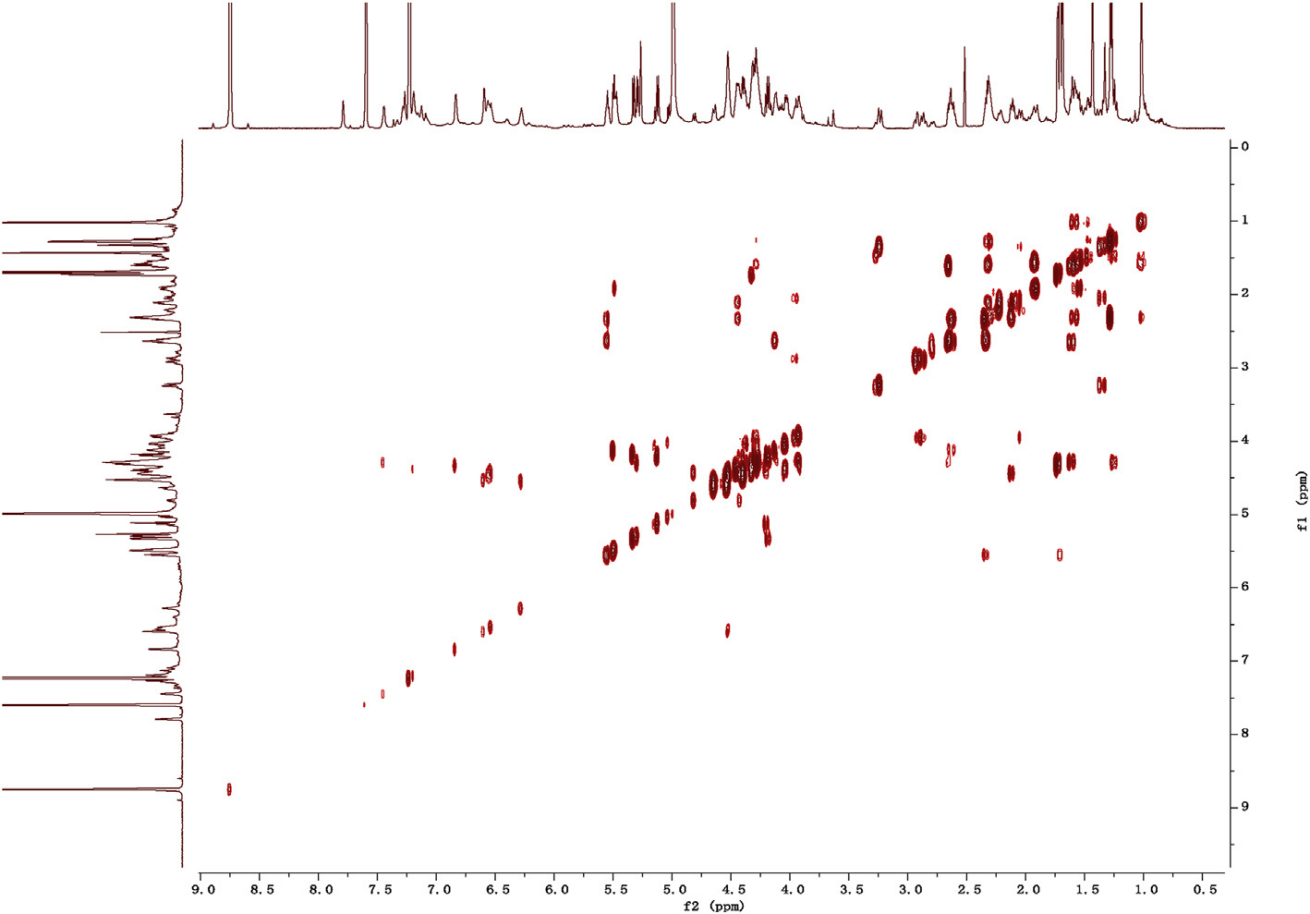
2D COSY NMR spectrum in pyridine-*d*_5_ of 1.

**Fig. 12:**
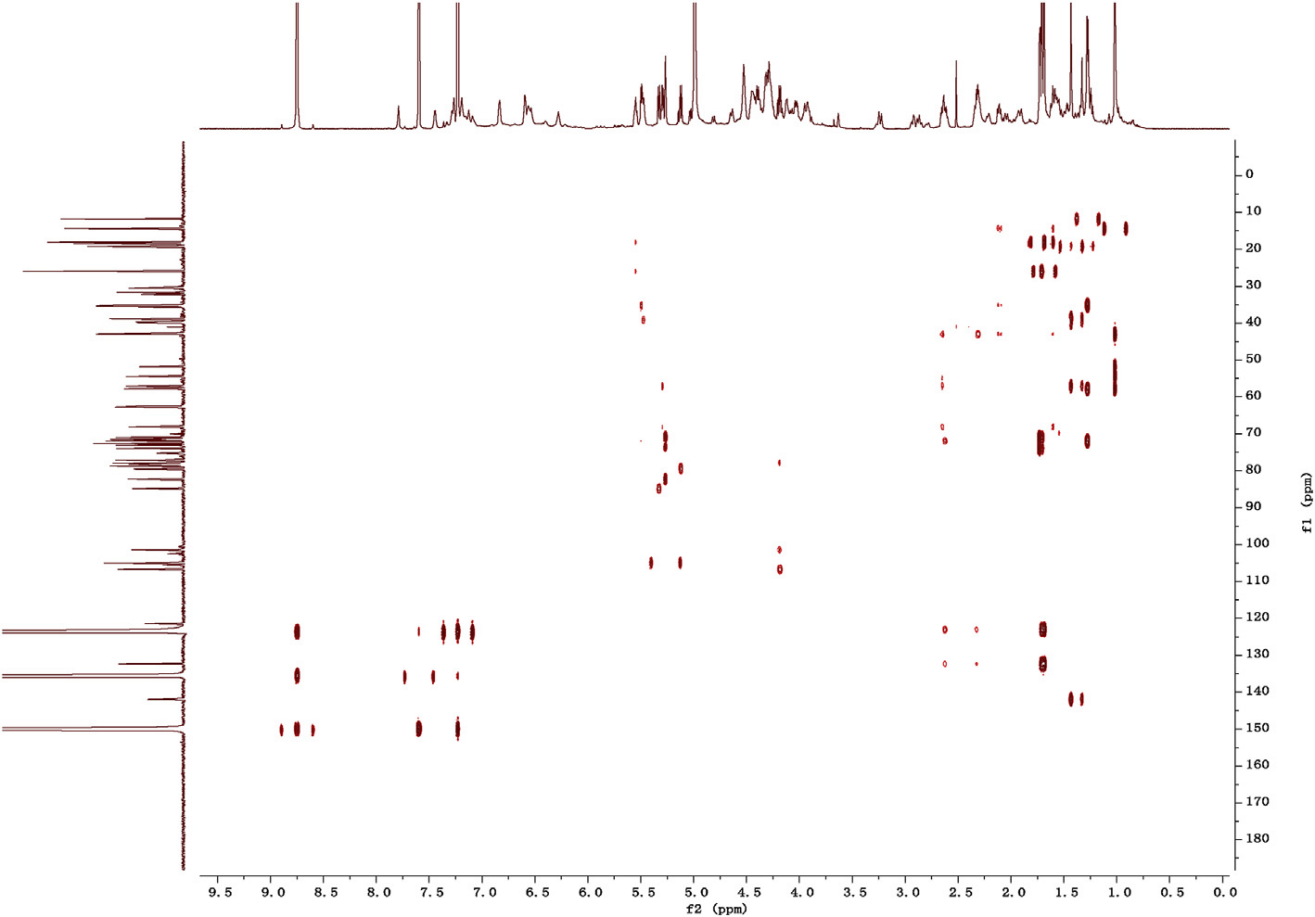
2D HMBC NMR spectrum in pyridine-*d*_5_ of 1.

**Fig. 13:**
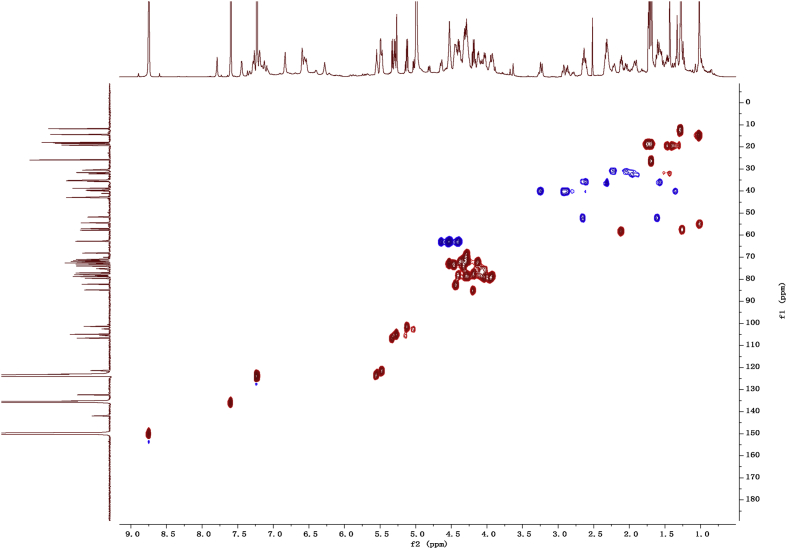
2D HSQC NMR spectrum in pyridine-*d*_5_ of 1.

**Fig. 14:**
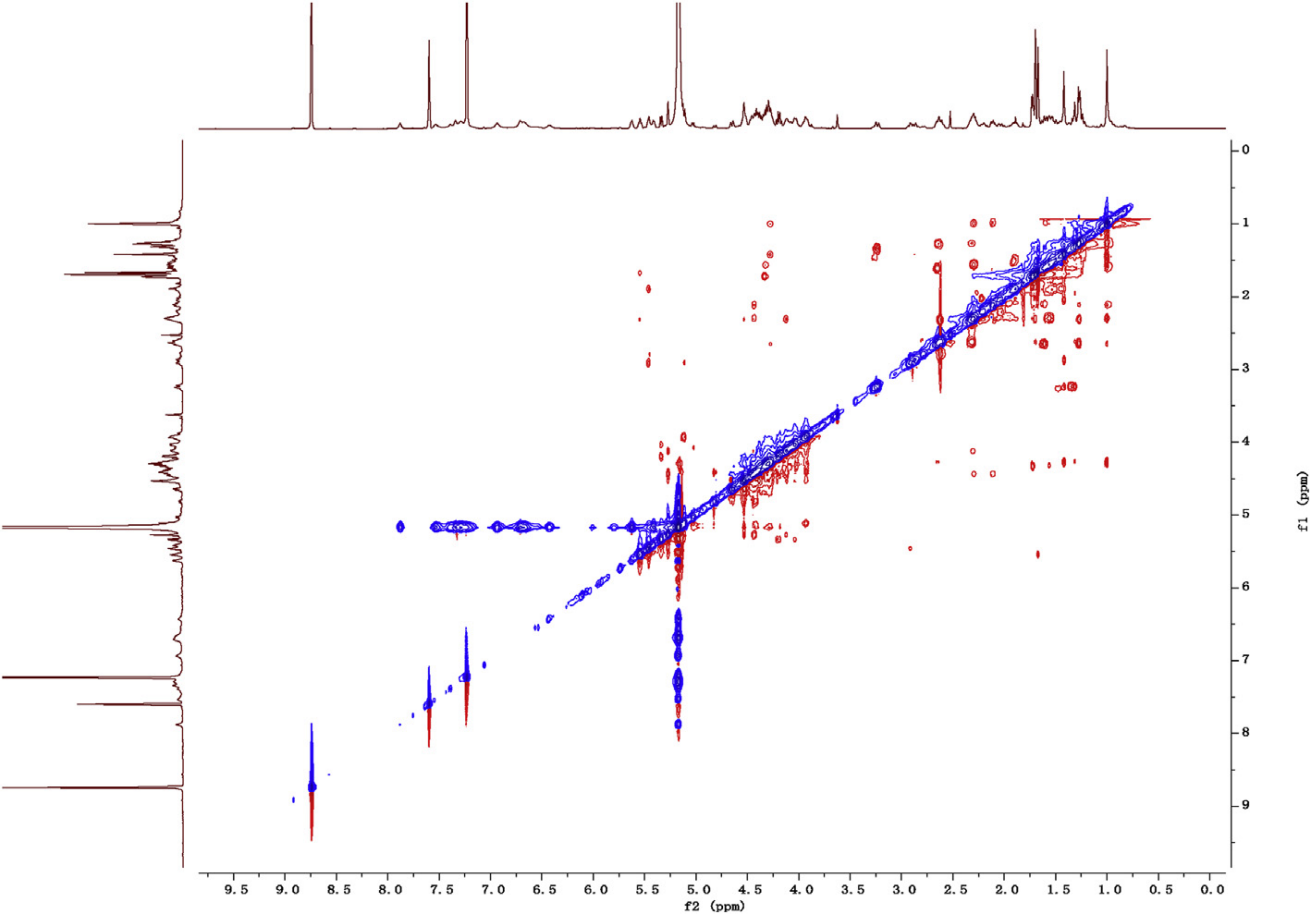
2D ROESY NMR spectrum in pyridine-*d*_5_ of 1.

**Fig. 15:**
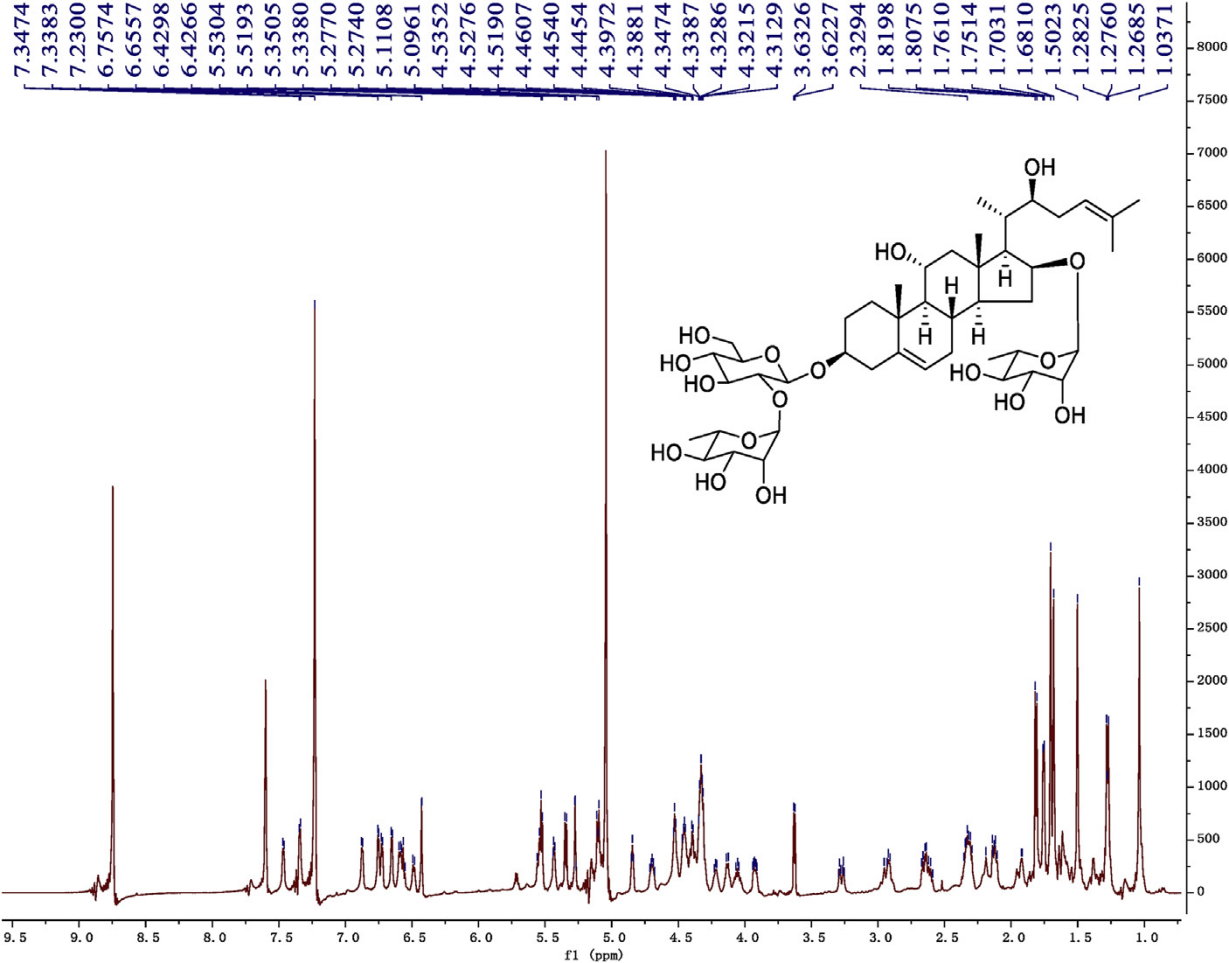
^1^H-NMR (500 MHz) spectrum in pyridine-*d*_5_ of 2.

**Fig. 16:**
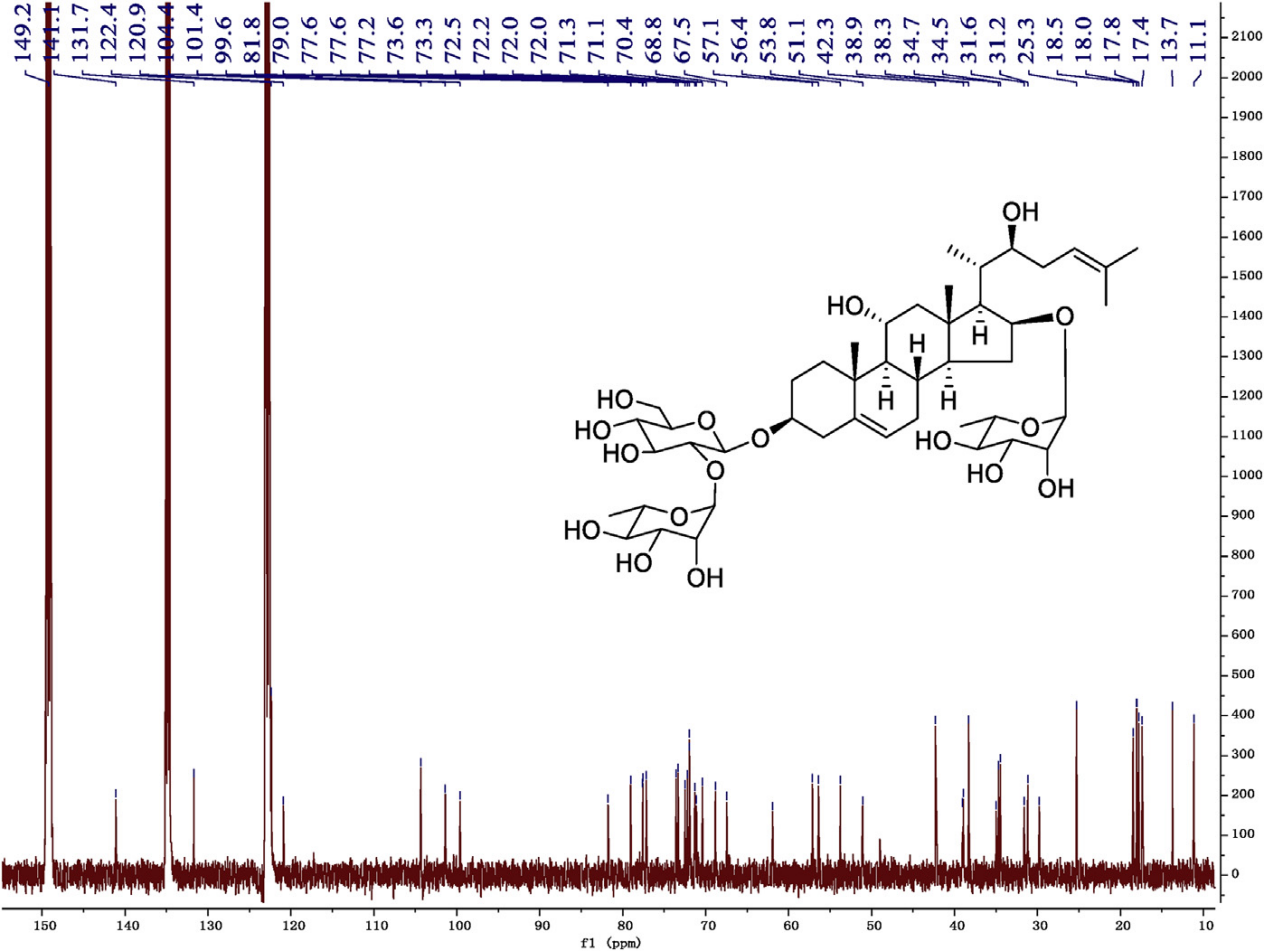
^13^C -NMR (125 MHz)spectrum in pyridine-*d*_5_ of 2.

**Fig. 17:**
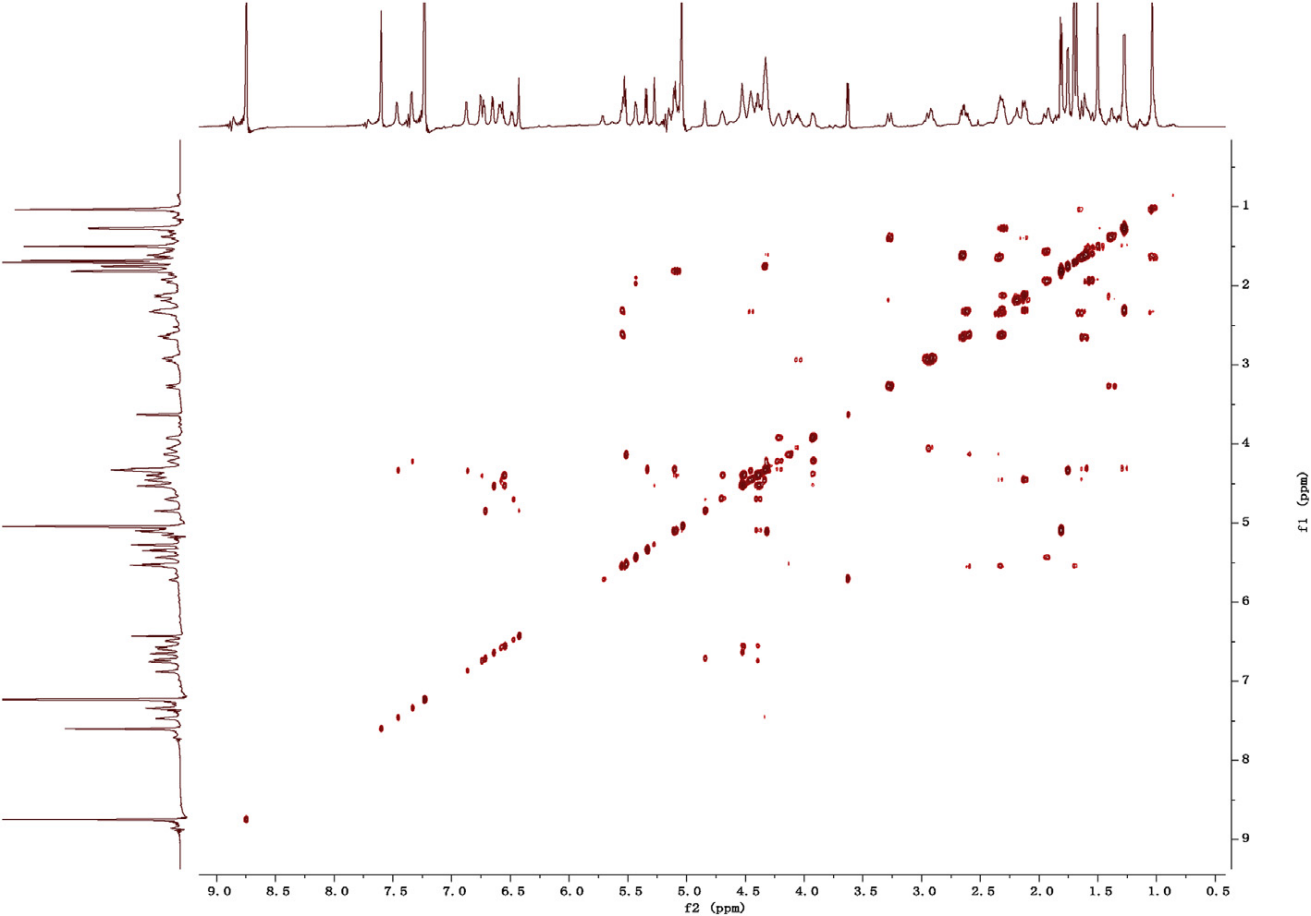
2D COSY NMR spectrum in pyridine-*d*_5_ of 2.

**Fig. 18:**
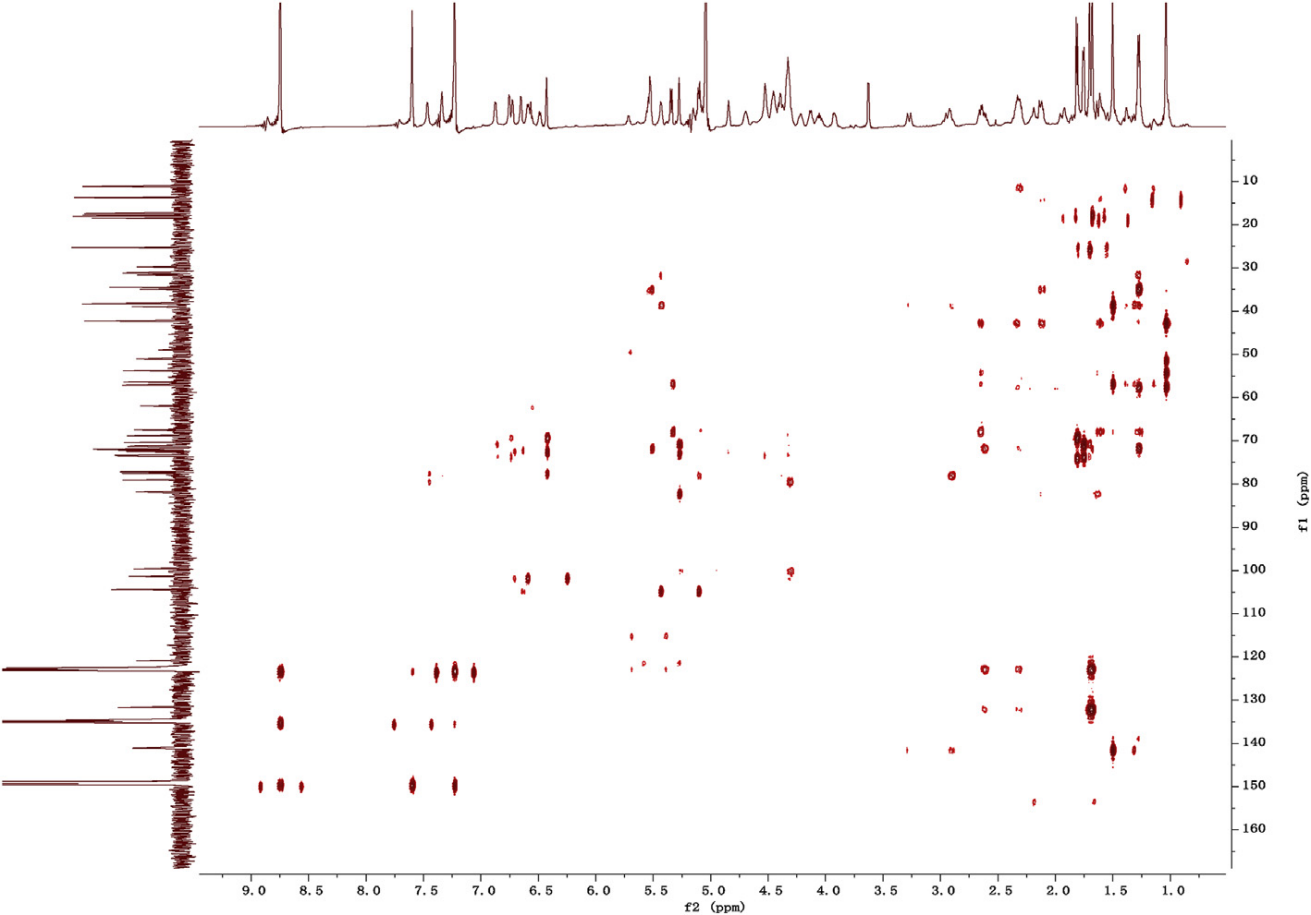
2D HMBC NMR spectrum in pyridine-*d*_5_ of 2.

**Fig. 19:**
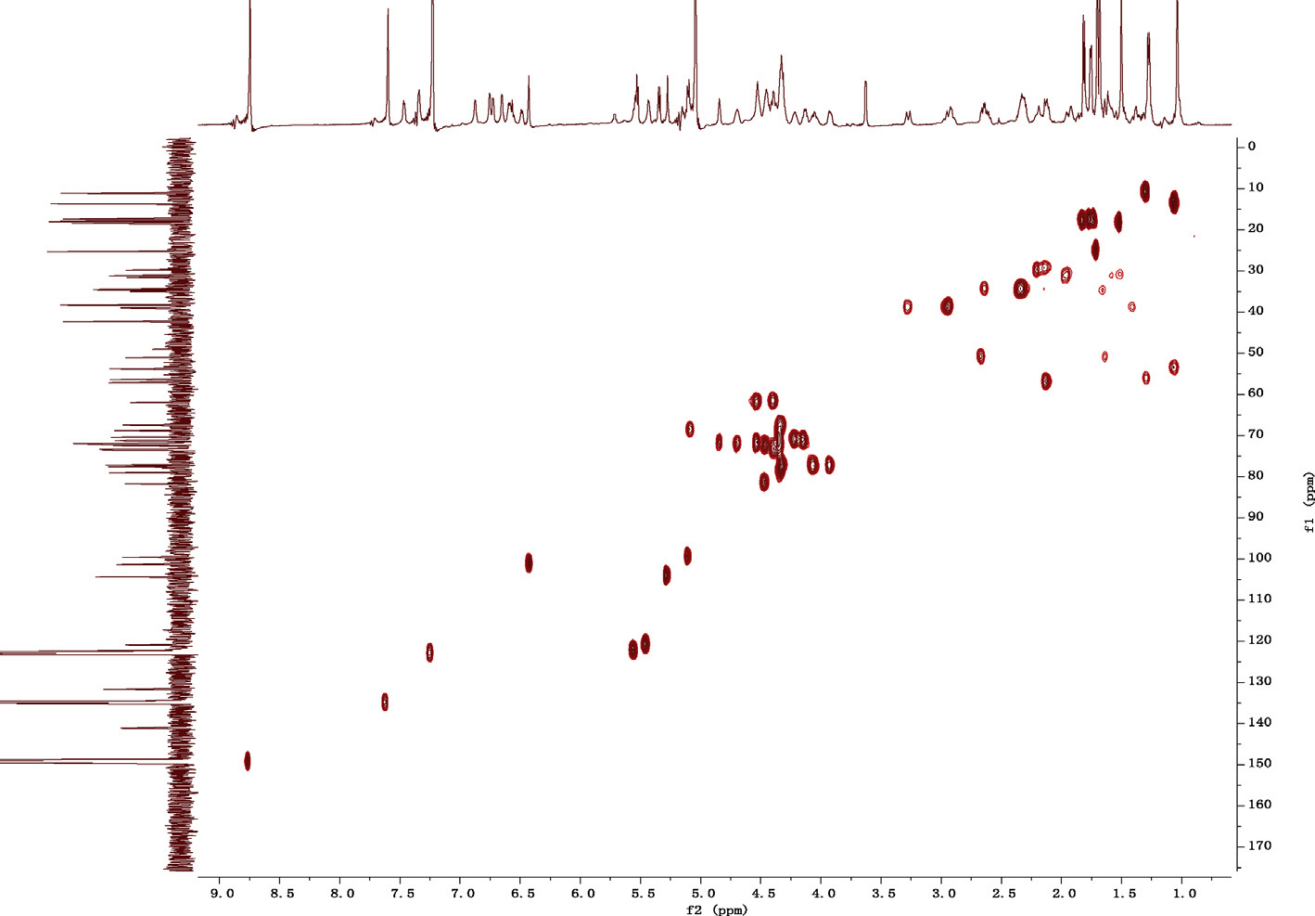
2D HSQC NMR spectrum in pyridine-*d*_5_ of 2.

**Fig. 20:**
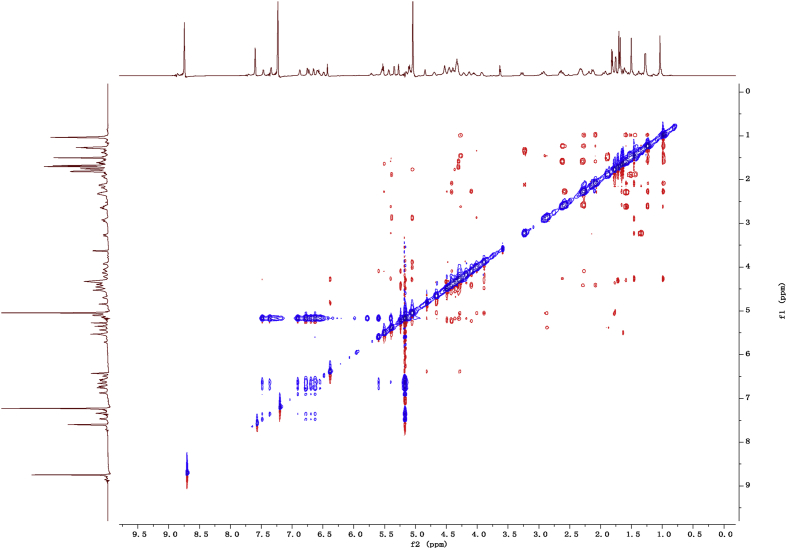
2D ROESY NMR spectrum in pyridine-*d*_5_ of 2.

**Fig. 21:**
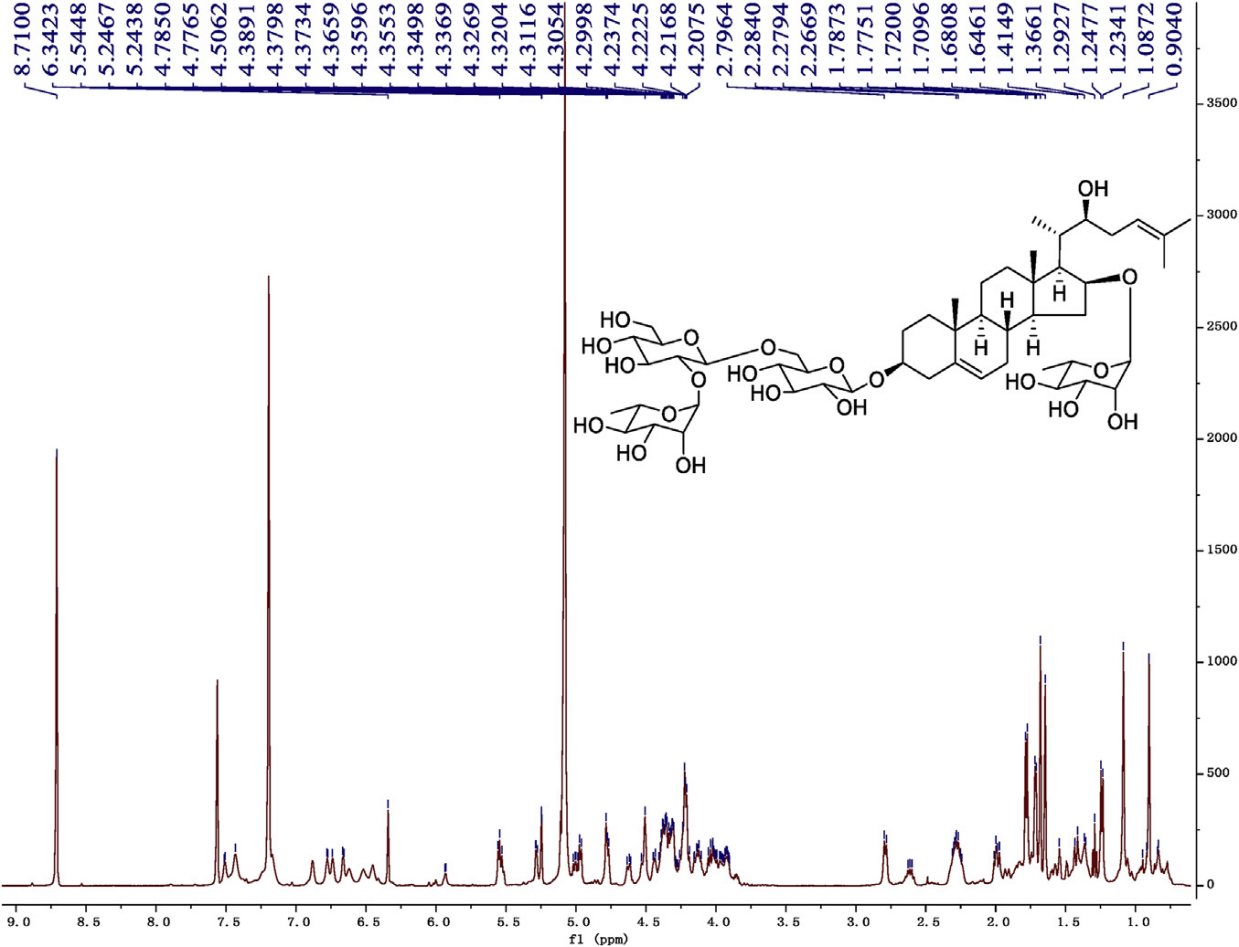
^1^H-NMR (500 MHz) spectrum in pyridine-*d*_5_ of 3.

**Fig. 22:**
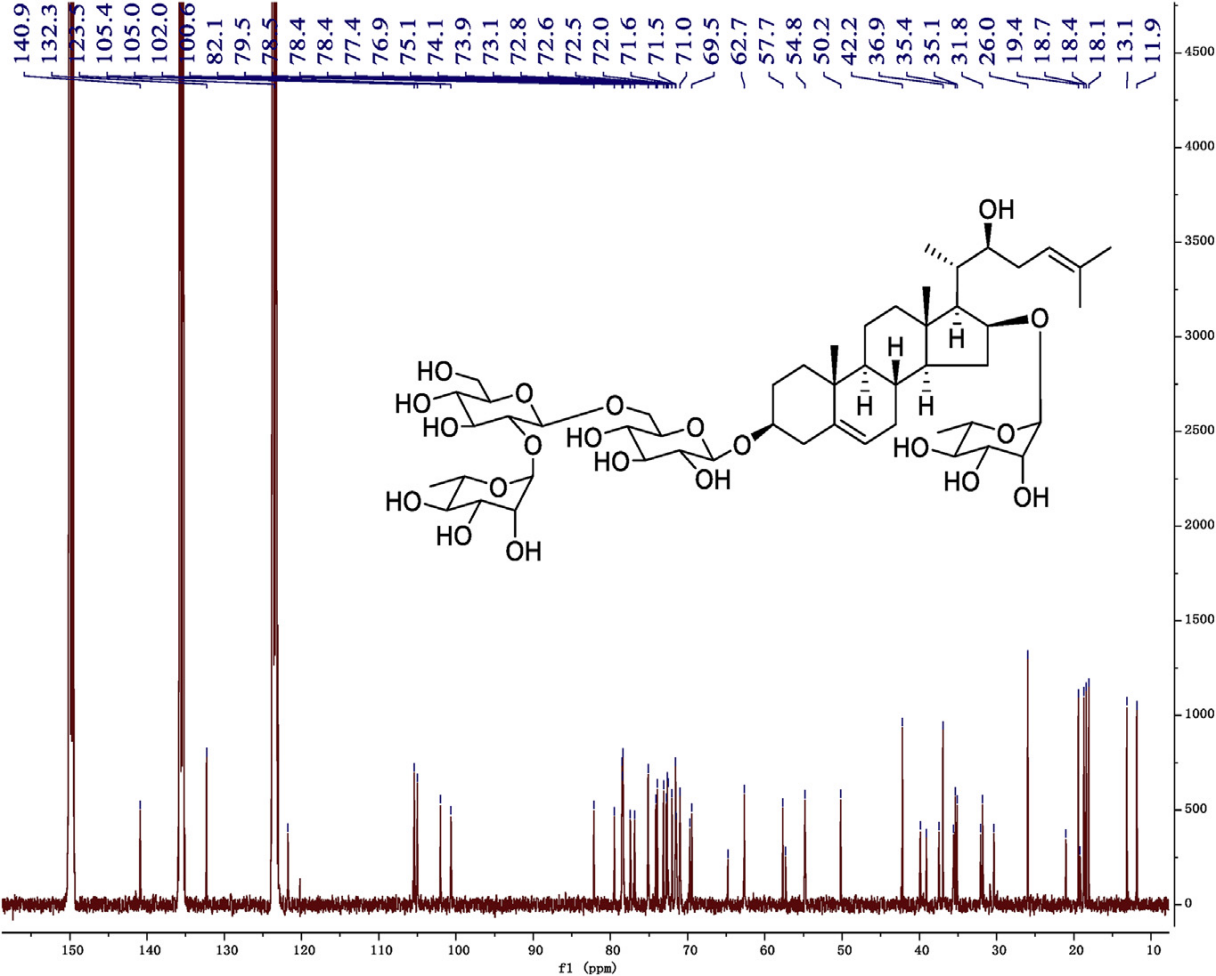
^13^C -NMR (125 MHz) spectrum in pyridine-*d*_5_ of 3.

**Fig. 23:**
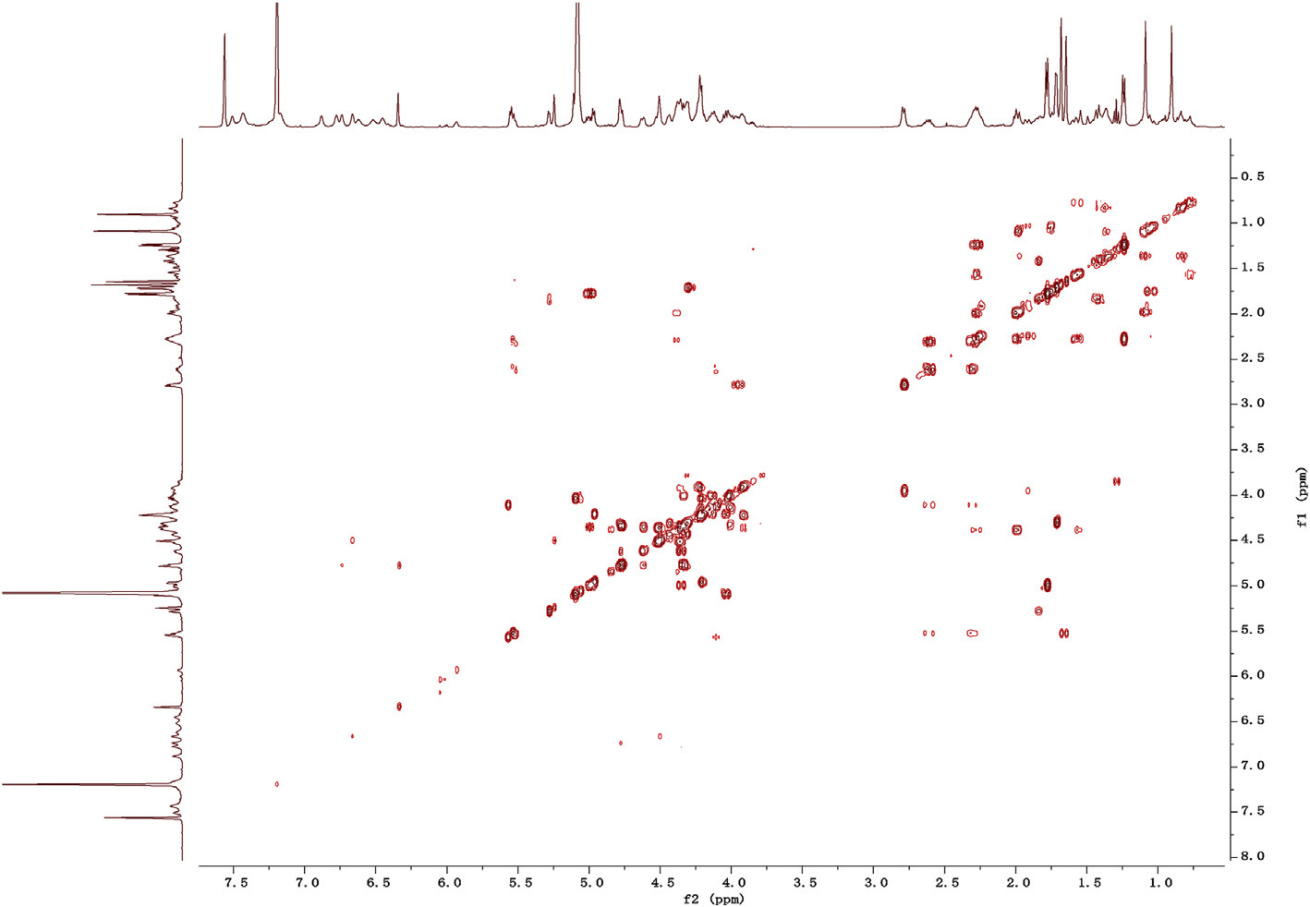
2D COSY NMR spectrum in pyridine-*d*_5_ of 3.

**Fig. 24:**
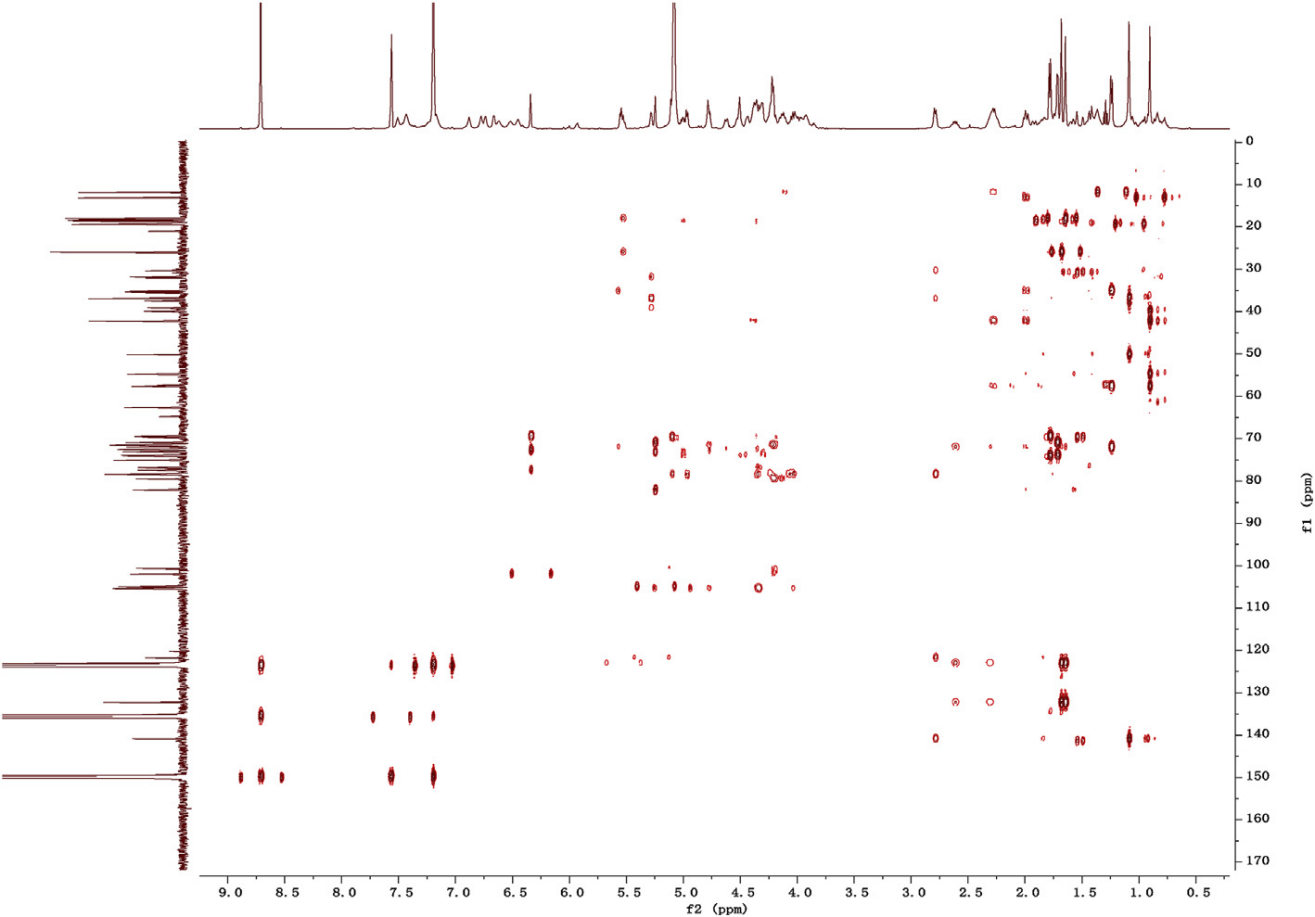
2D HMBC NMR spectrum in pyridine-*d*_5_ of 3.

**Fig. 25:**
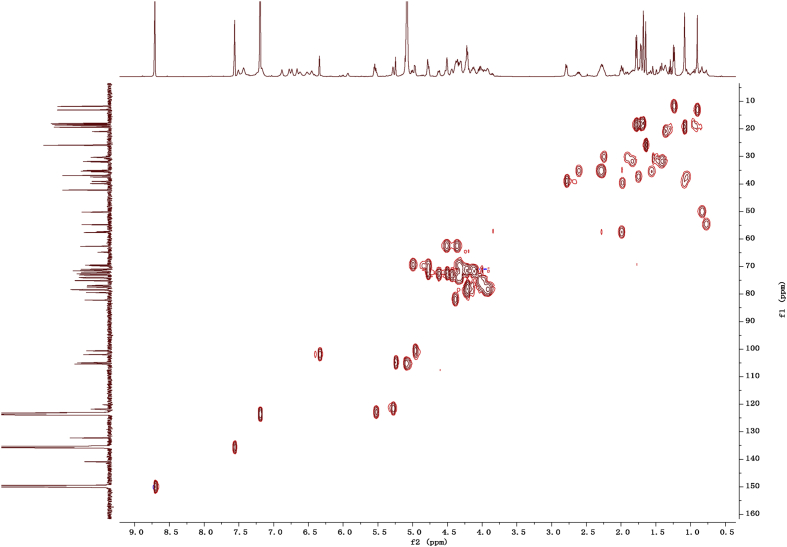
2D HSQC NMR spectrum in pyridine-*d*_5_ of 3.

**Fig. 26:**
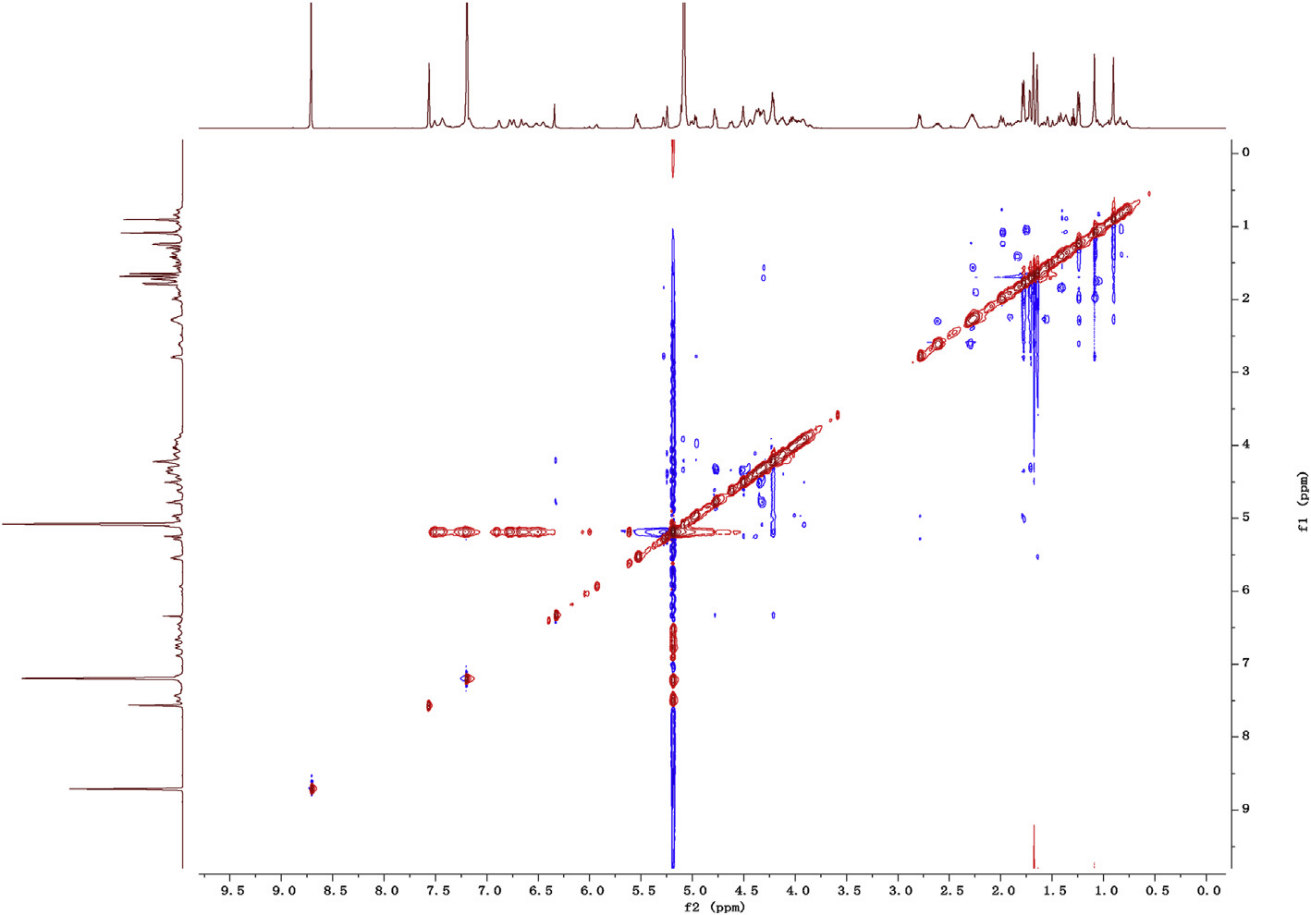
2D ROESY NMR spectrum in pyridine-*d*_5_ of 3.

**Fig. 27:**
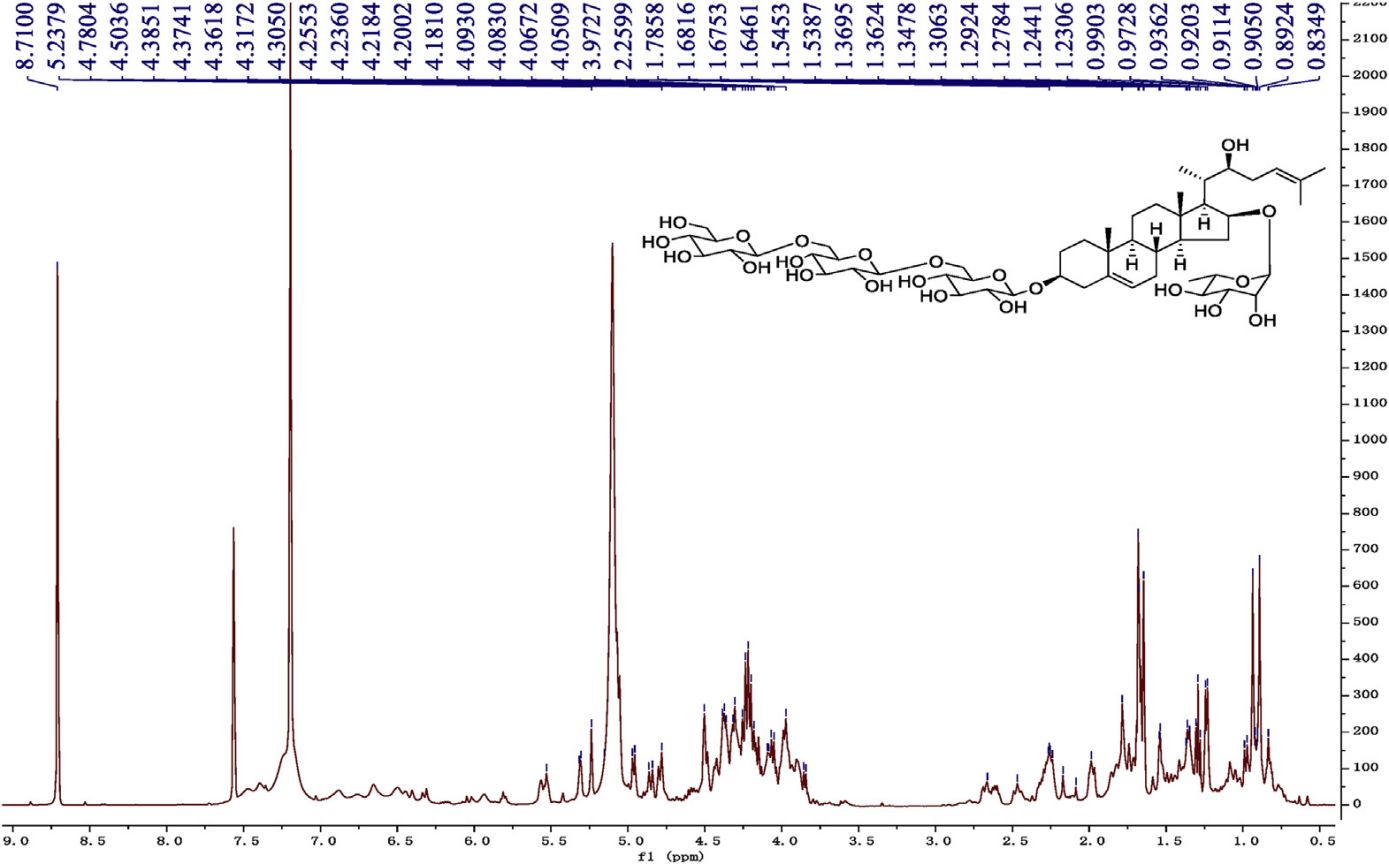
^1^H-NMR (500 MHz) spectrum in pyridine-*d*_5_ of 4.

**Fig. 28:**
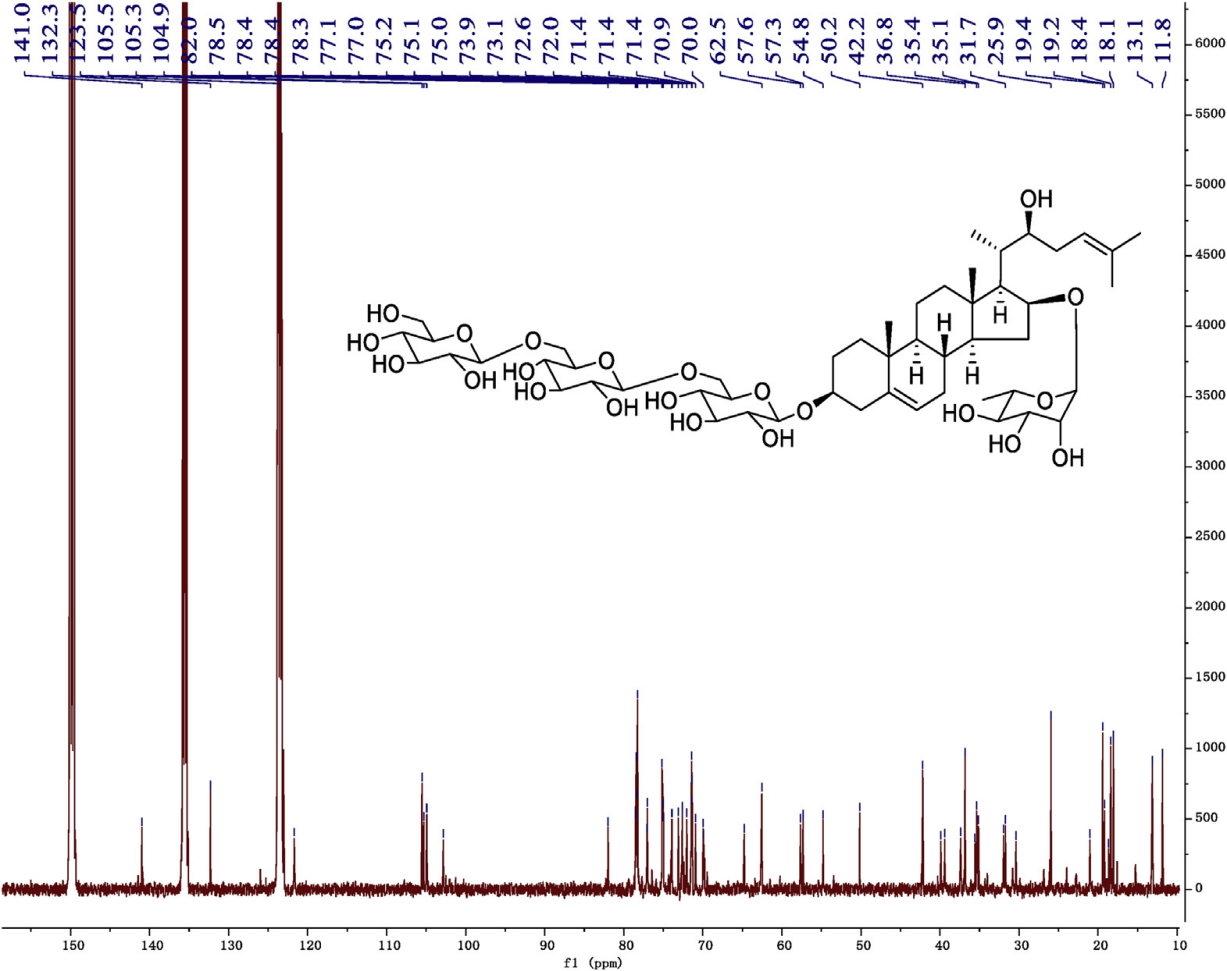
^13^C -NMR (125 MHz) spectrum in pyridine-*d*_5_ of 4.

**Fig. 29:**
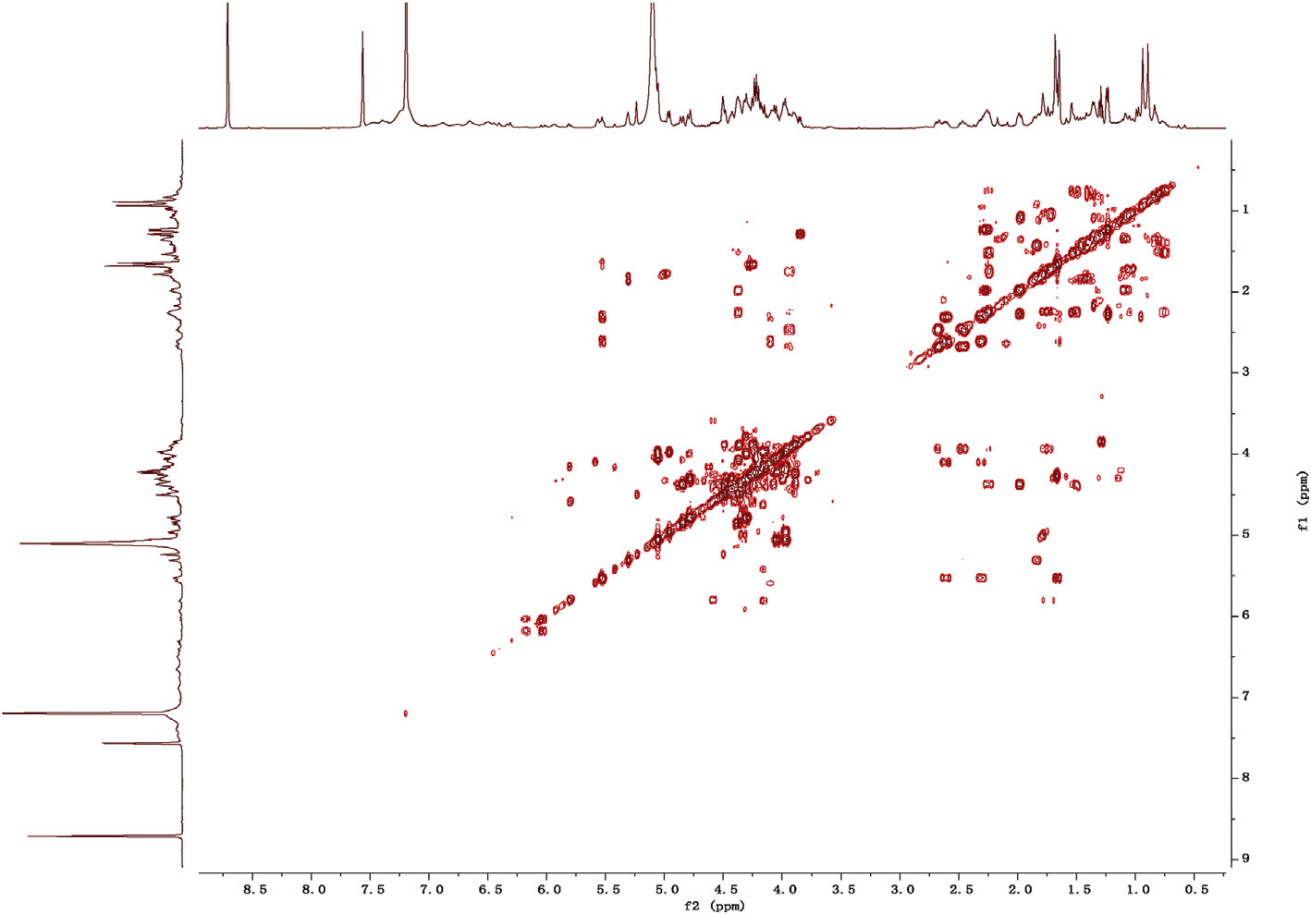
2D COSY NMR spectrum in pyridine-*d*_5_ of 4.

**Fig. 30:**
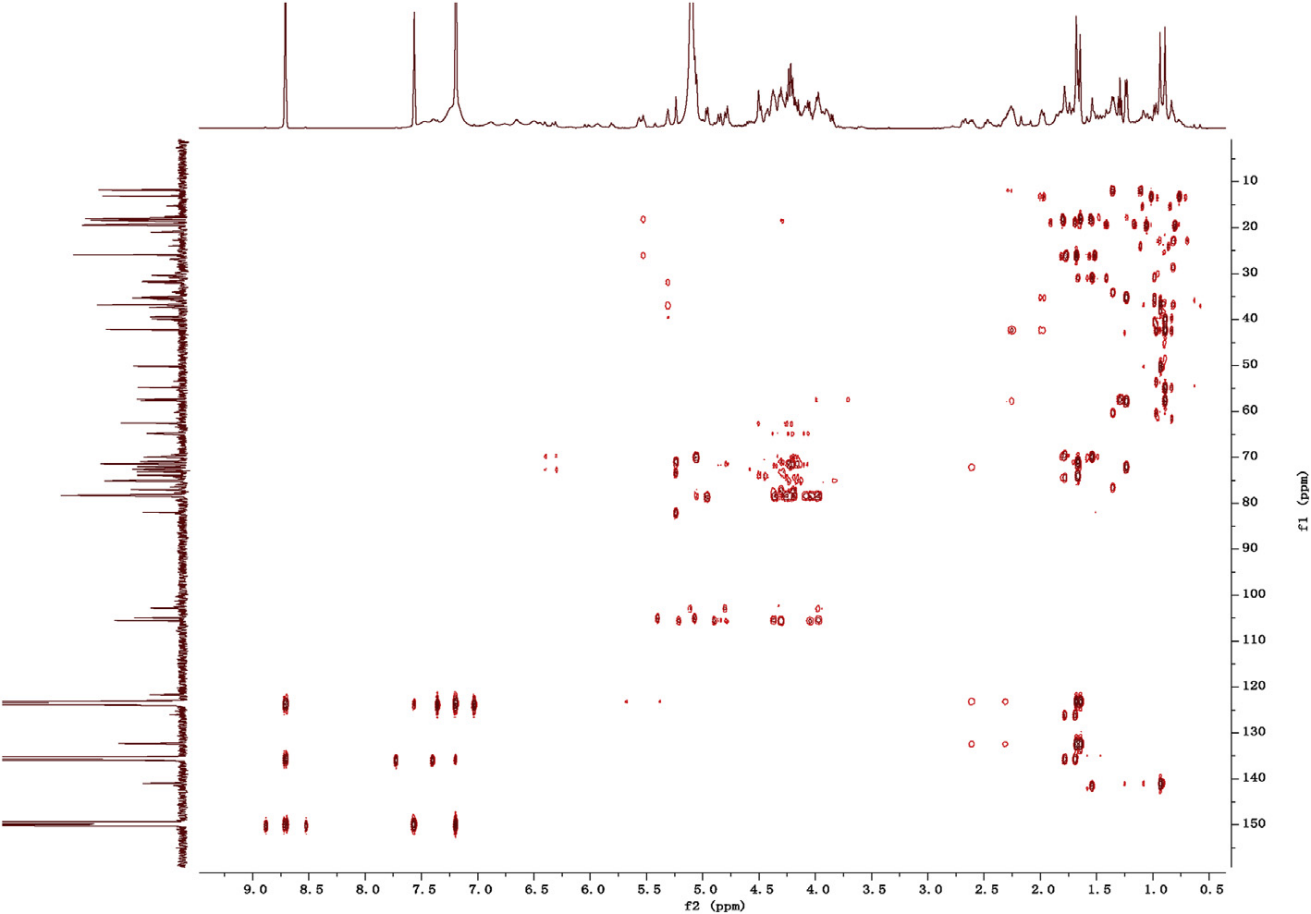
2D HMBC NMR spectrum in pyridine-*d*_5_ of 4.

**Fig. 31:**
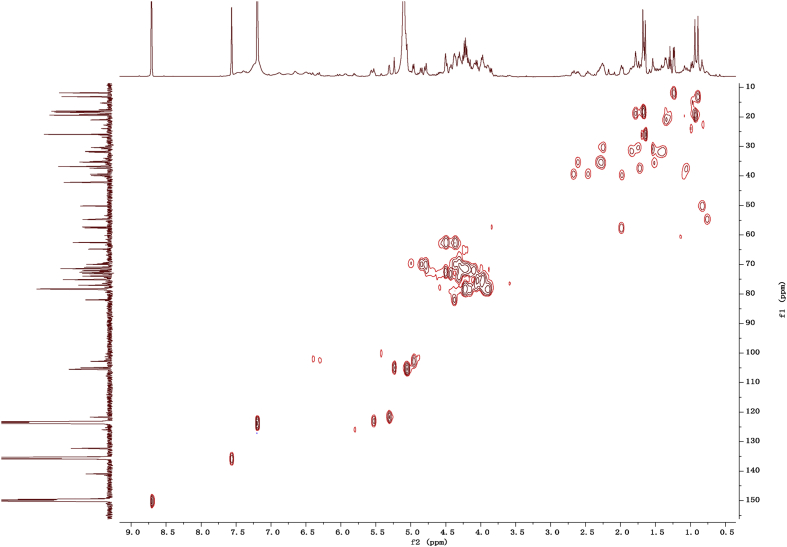
2D HSQC NMR spectrum in pyridine-*d*_5_ of 4.

**Fig. 32:**
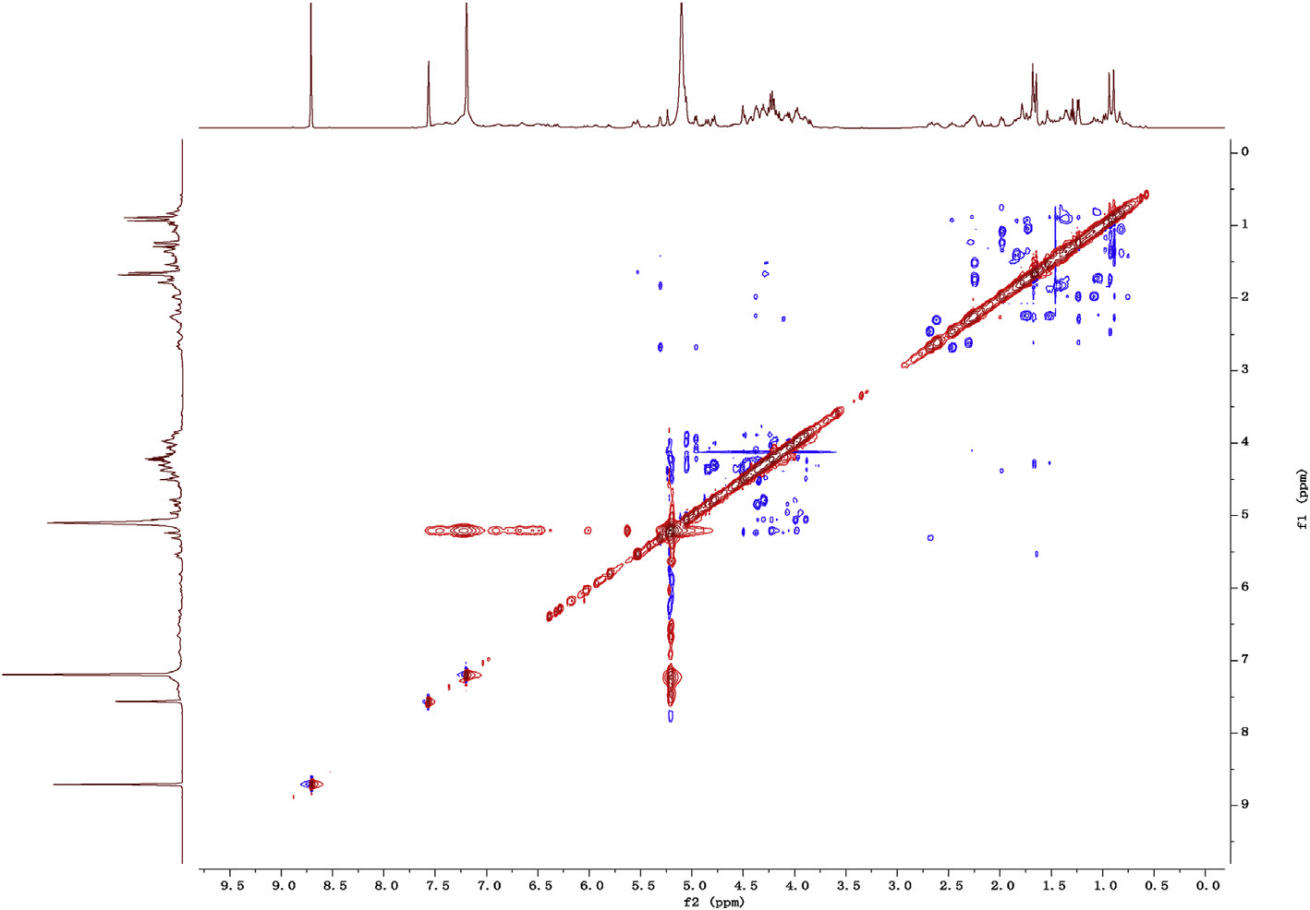
2D ROESY NMR spectrum in pyridine-*d*_5_ of 4.

**Fig. 33:**
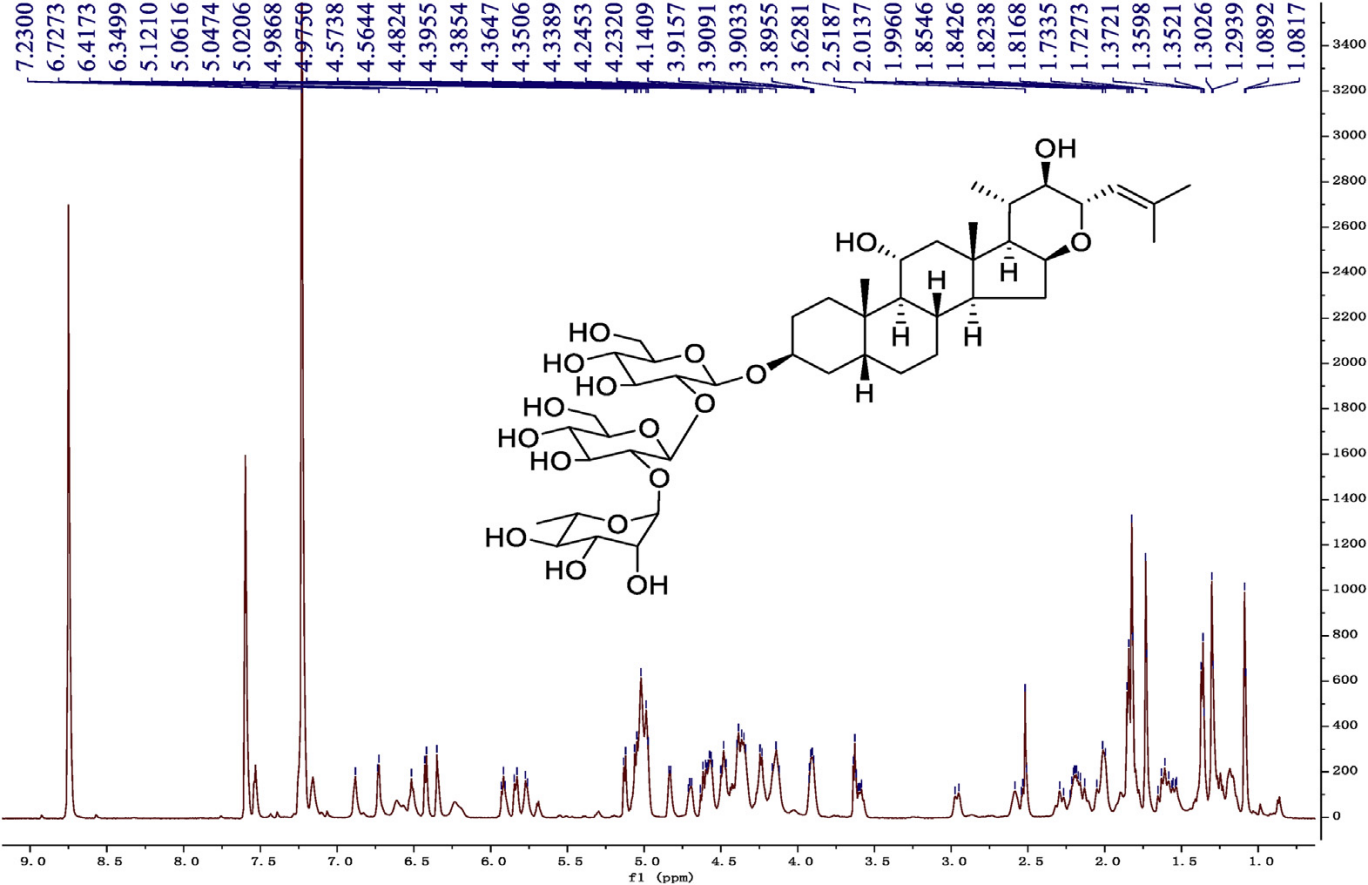
^1^H-NMR (500 MHz)spectrum in pyridine-*d*_5_ of 5.

**Fig. 34:**
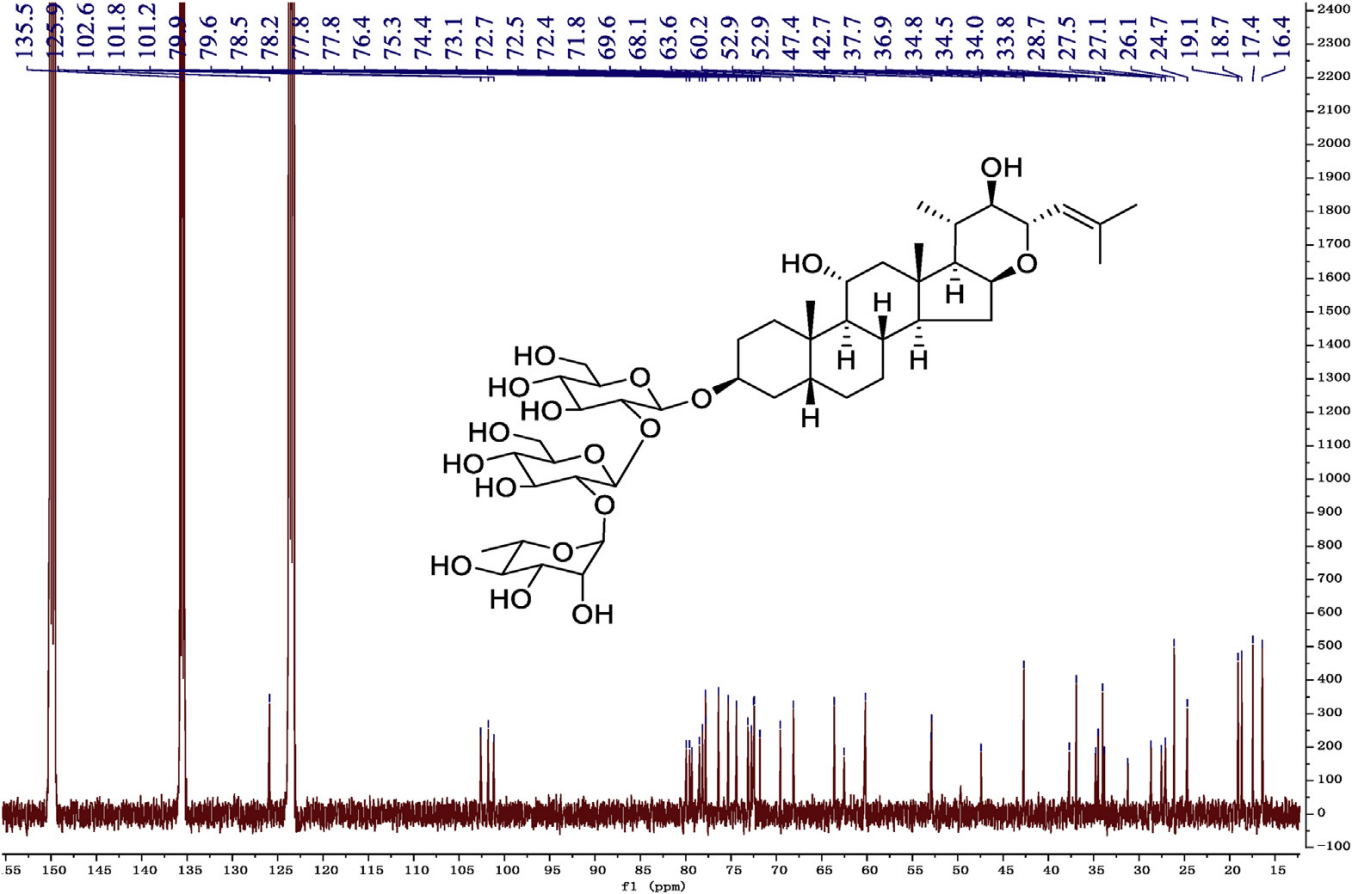
^13^C -NMR (125 MHz) spectrum in pyridine-*d*_5_ of 5.

**Fig. 35:**
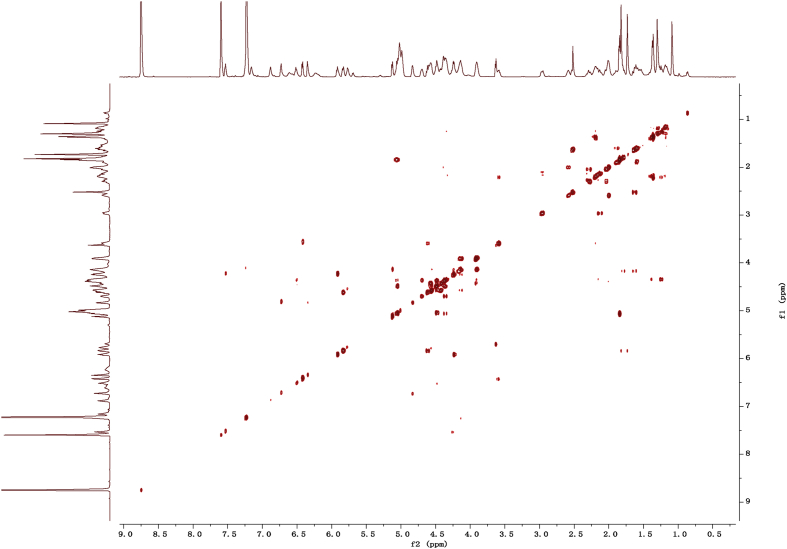
2D COSY NMR spectrum in pyridine-*d*_5_ of 5.

**Fig. 36:**
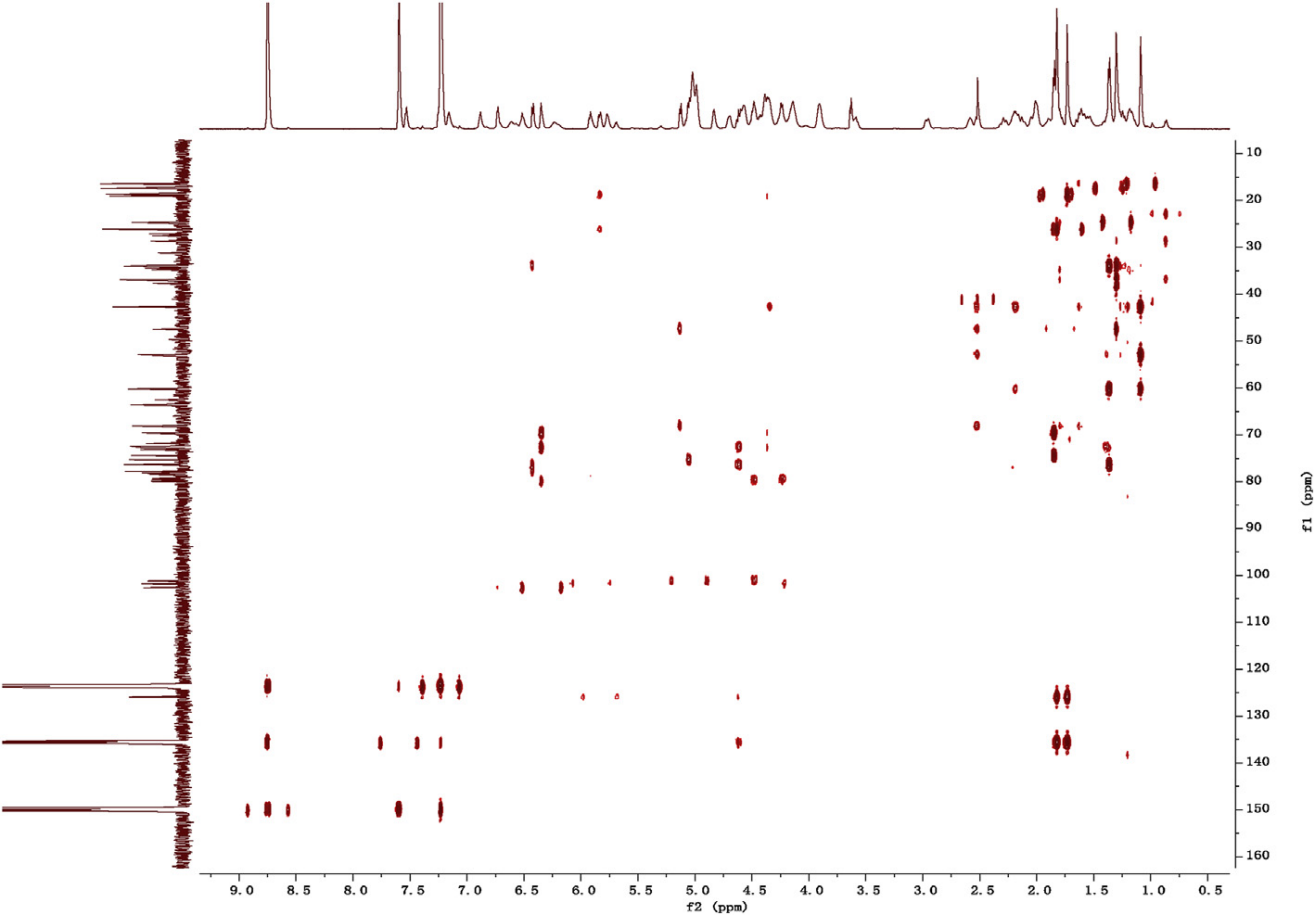
2D HMBC NMR spectrum in pyridine-*d*_5_ of 5.

**Fig. 37:**
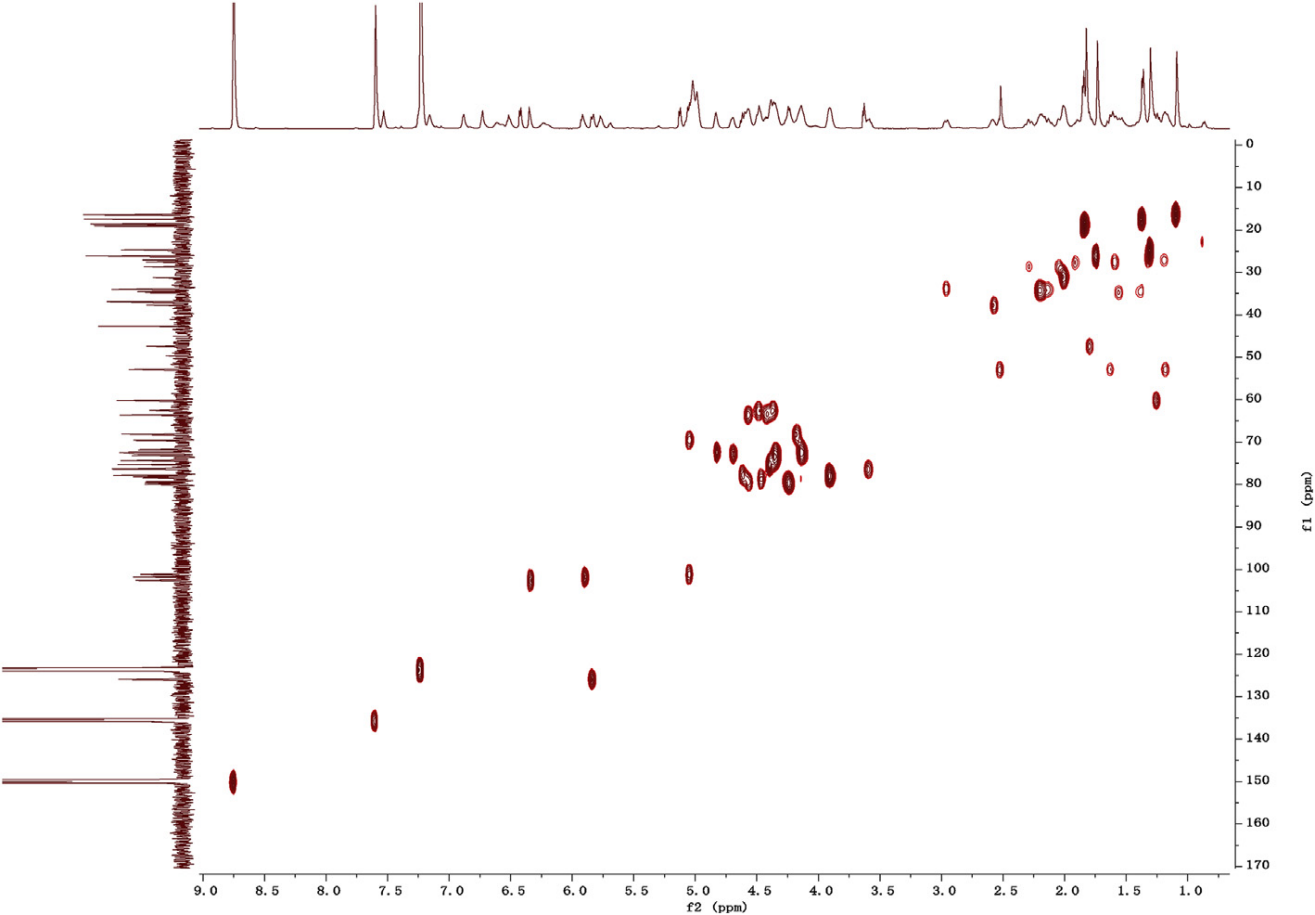
2D HSQC NMR spectrum in pyridine-*d*_5_ of 5.

**Fig. 38:**
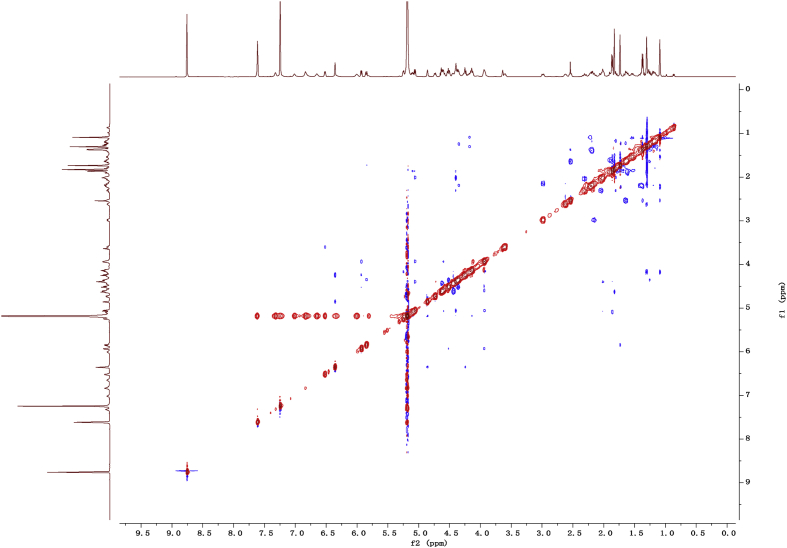
2D ROESY NMR spectrum in pyridine-*d*_5_ of 5.

**Fig. 39:**
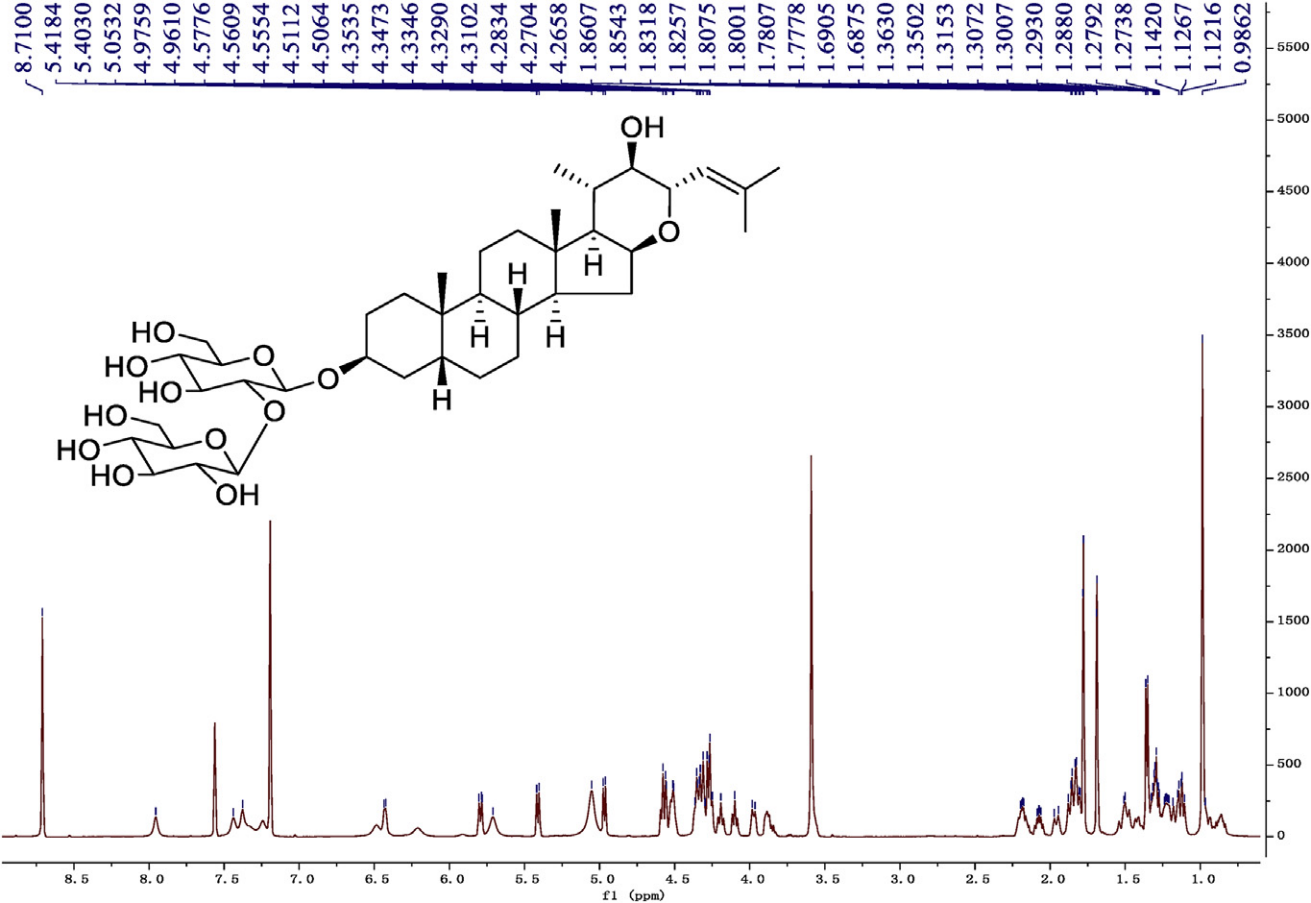
^1^H-NMR (500 MHz) spectrum in pyridine-*d*_5_ of 6.

**Fig. 40:**
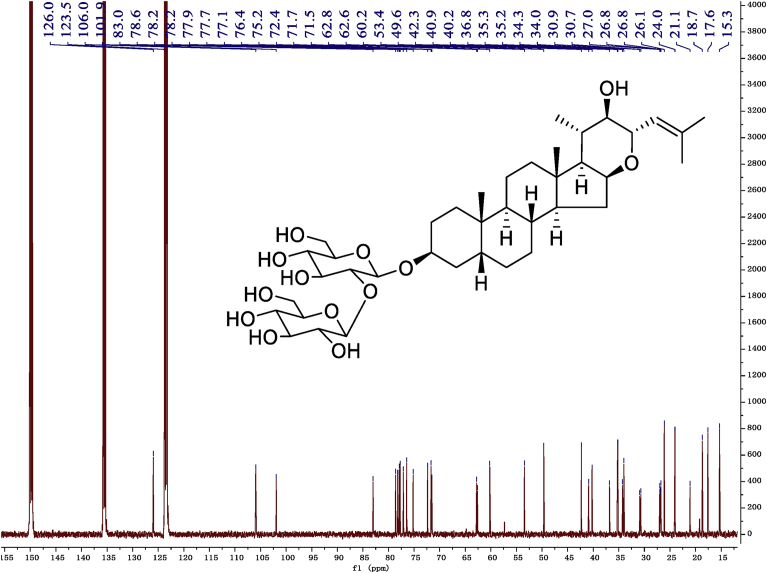
^13^C -NMR (125 MHz) spectrum in pyridine-*d*_5_ of 6.

**Fig. 41:**
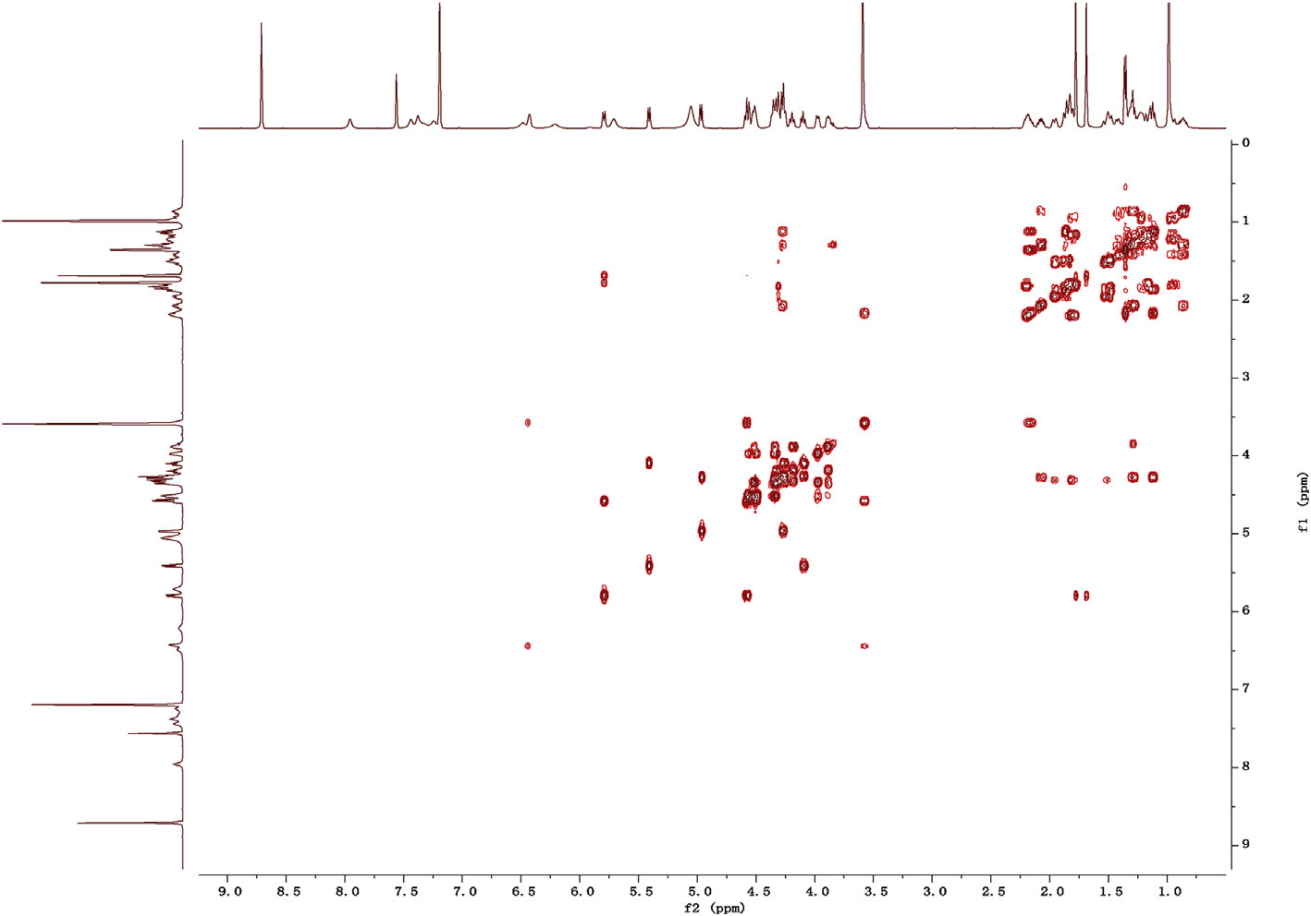
2D COSY NMR spectrum in pyridine-*d*_5_ of 6.

**Fig. 42:**
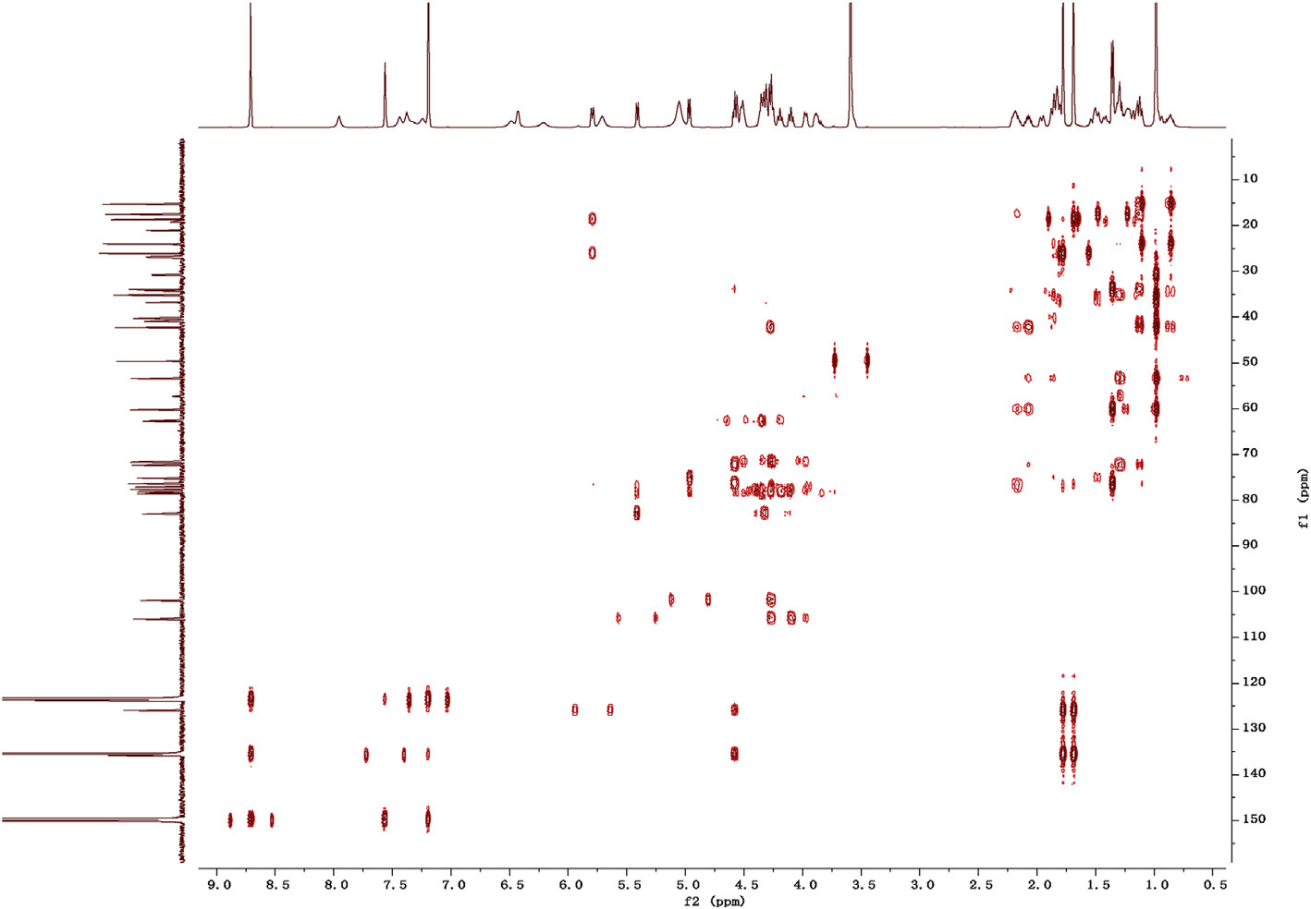
2D HMBC NMR spectrum in pyridine-*d*_5_ of 6.

**Fig. 43:**
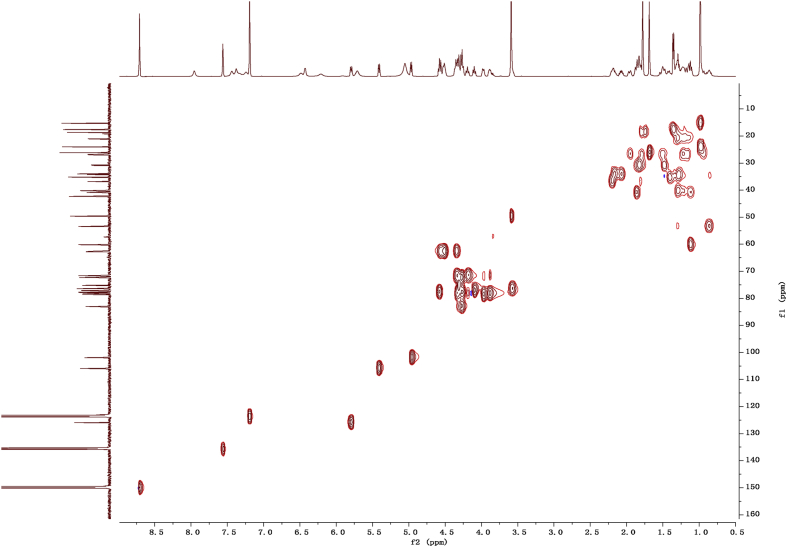
2D HSQC NMR spectrum in pyridine-*d*_5_ of 6.

**Fig. 44:**
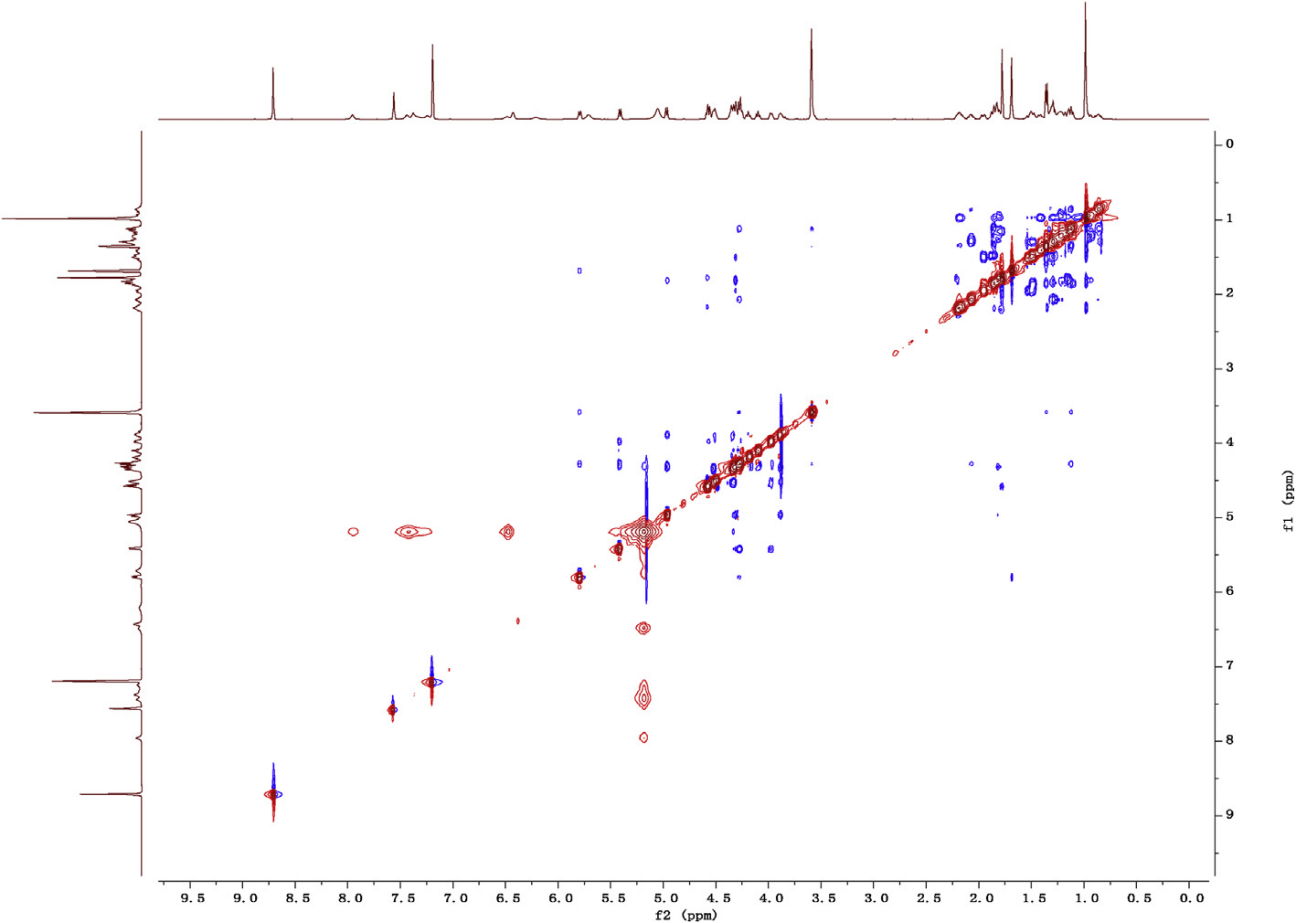
2D ROESY NMR spectrum in pyridine-*d*_5_ of 6.

**Fig. 45:**
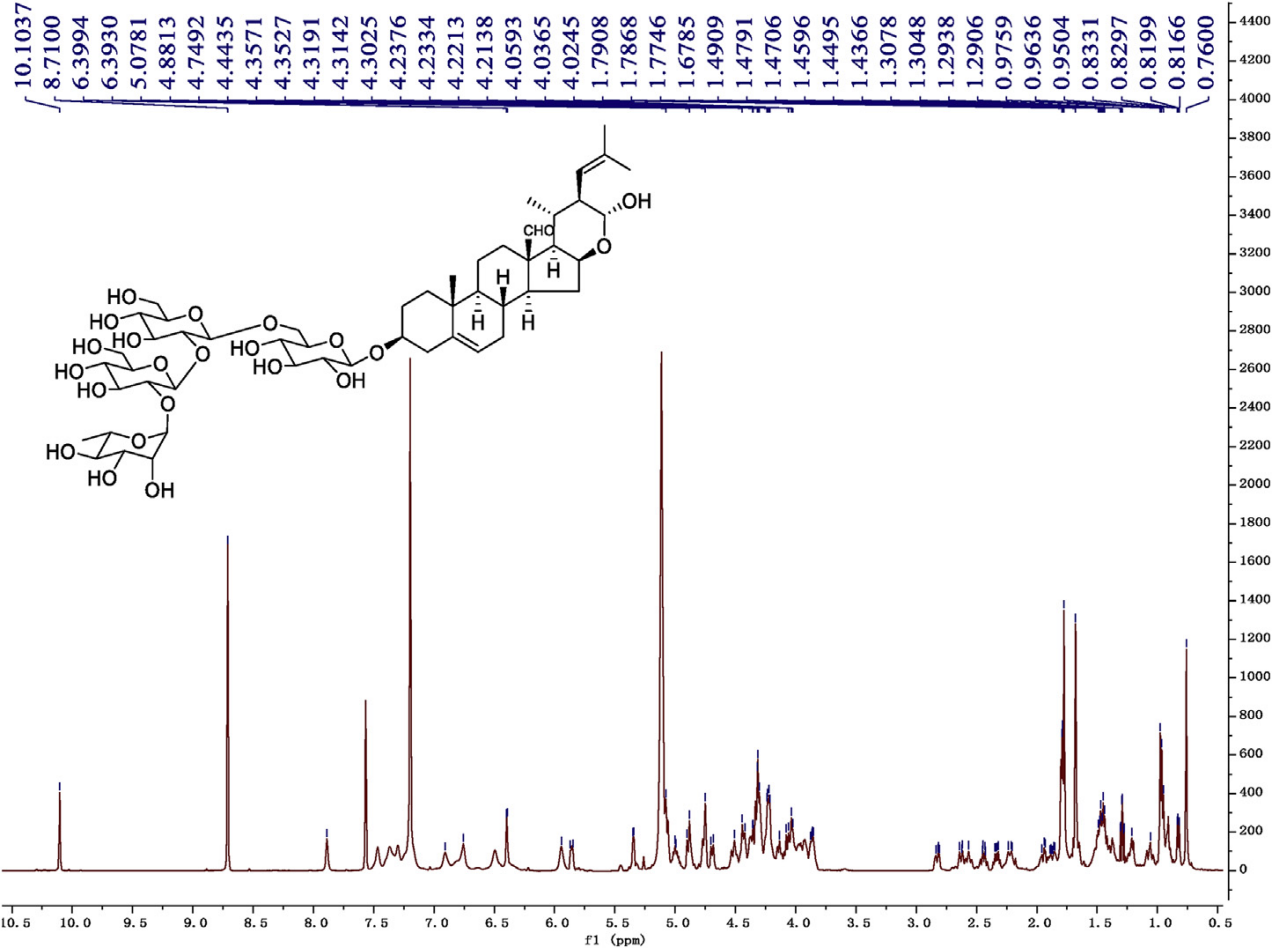
^1^H-NMR (500 MHz) spectrum in pyridine-*d*_5_ of 7.

**Fig. 46:**
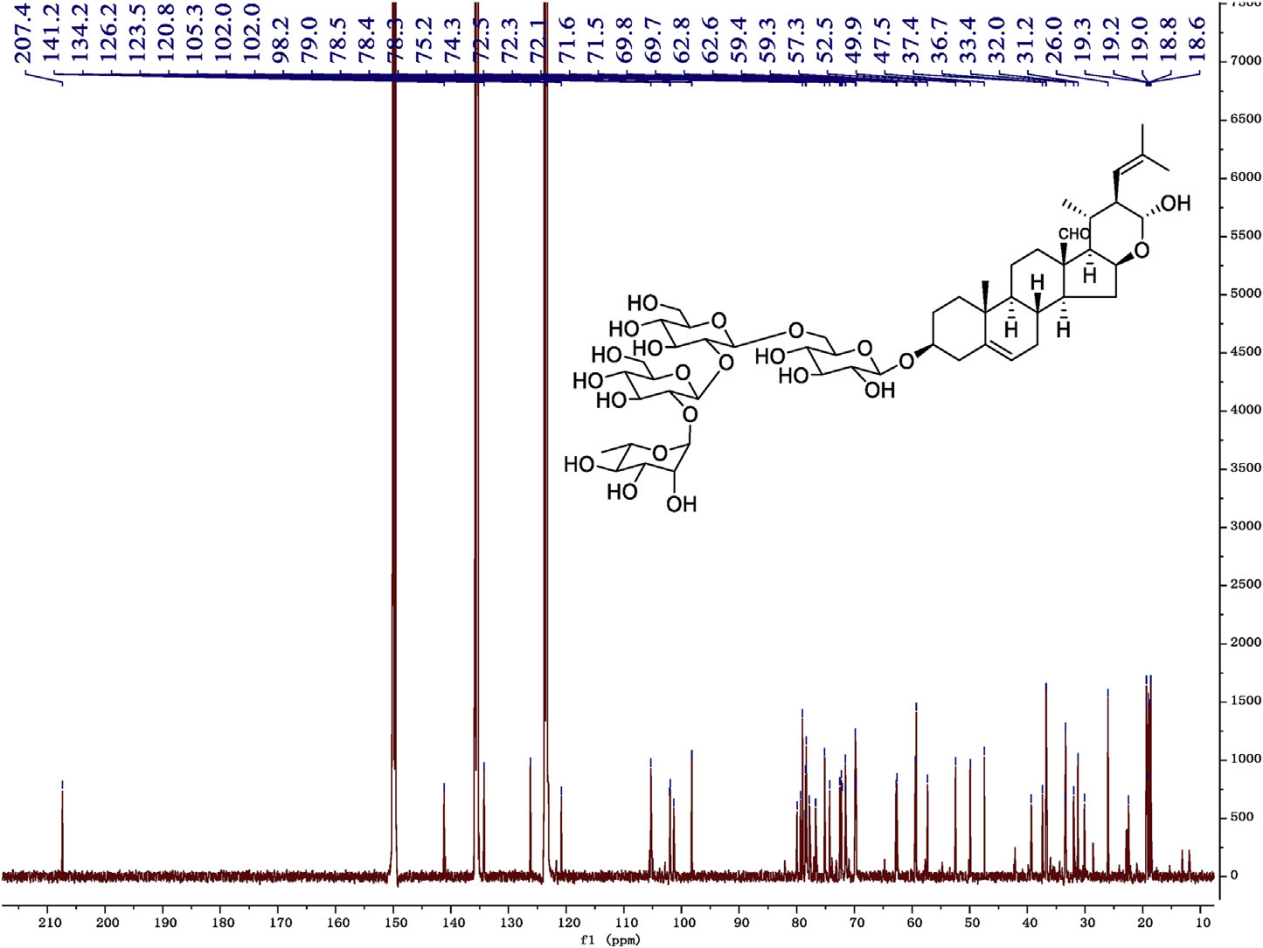
^1^C-NMR (125 MHz) spectrum in pyridine-*d*_5_ of 7.

**Fig. 47:**
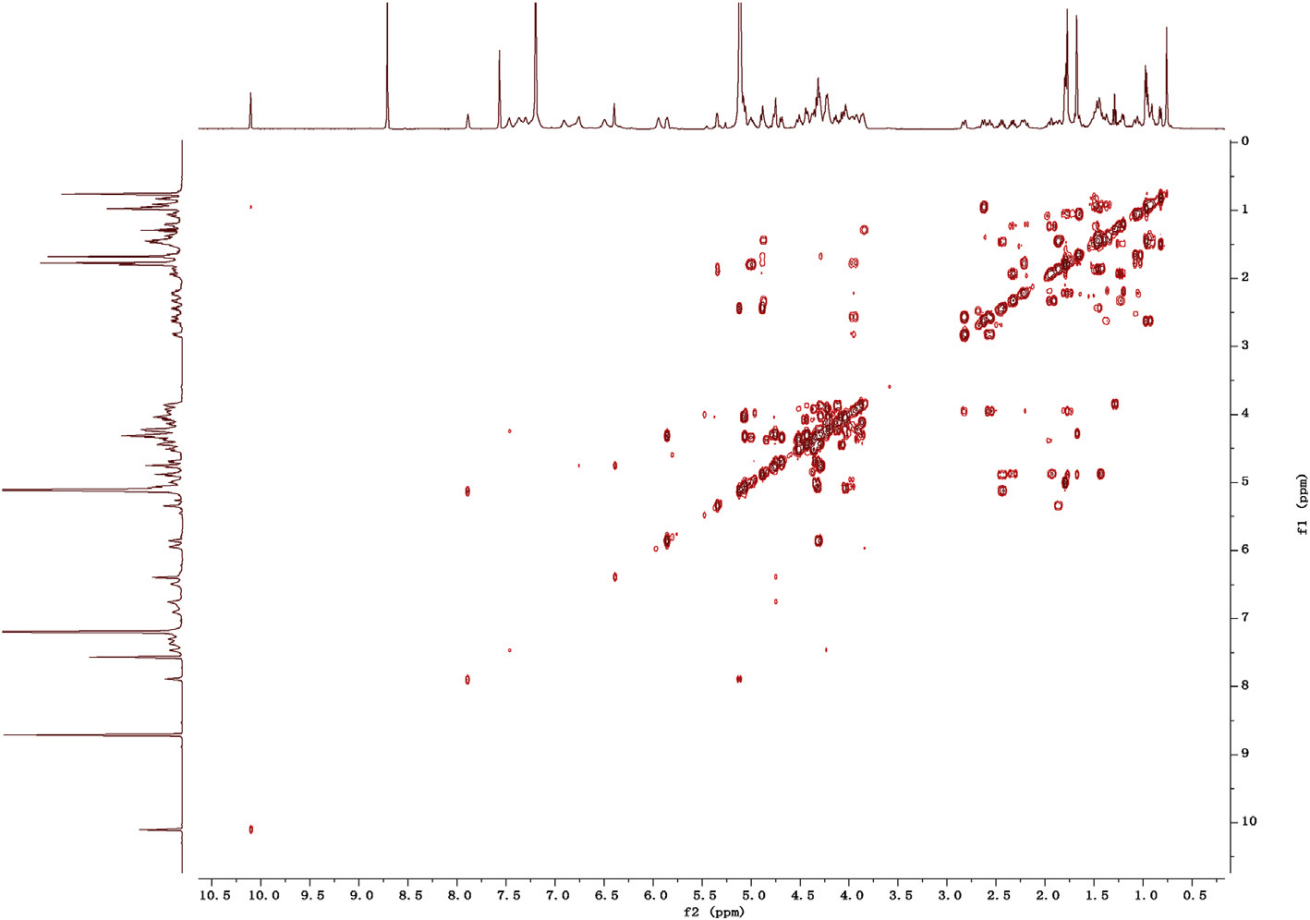
2D COSY NMR spectrum in pyridine-*d*_5_ of 7.

**Fig. 48:**
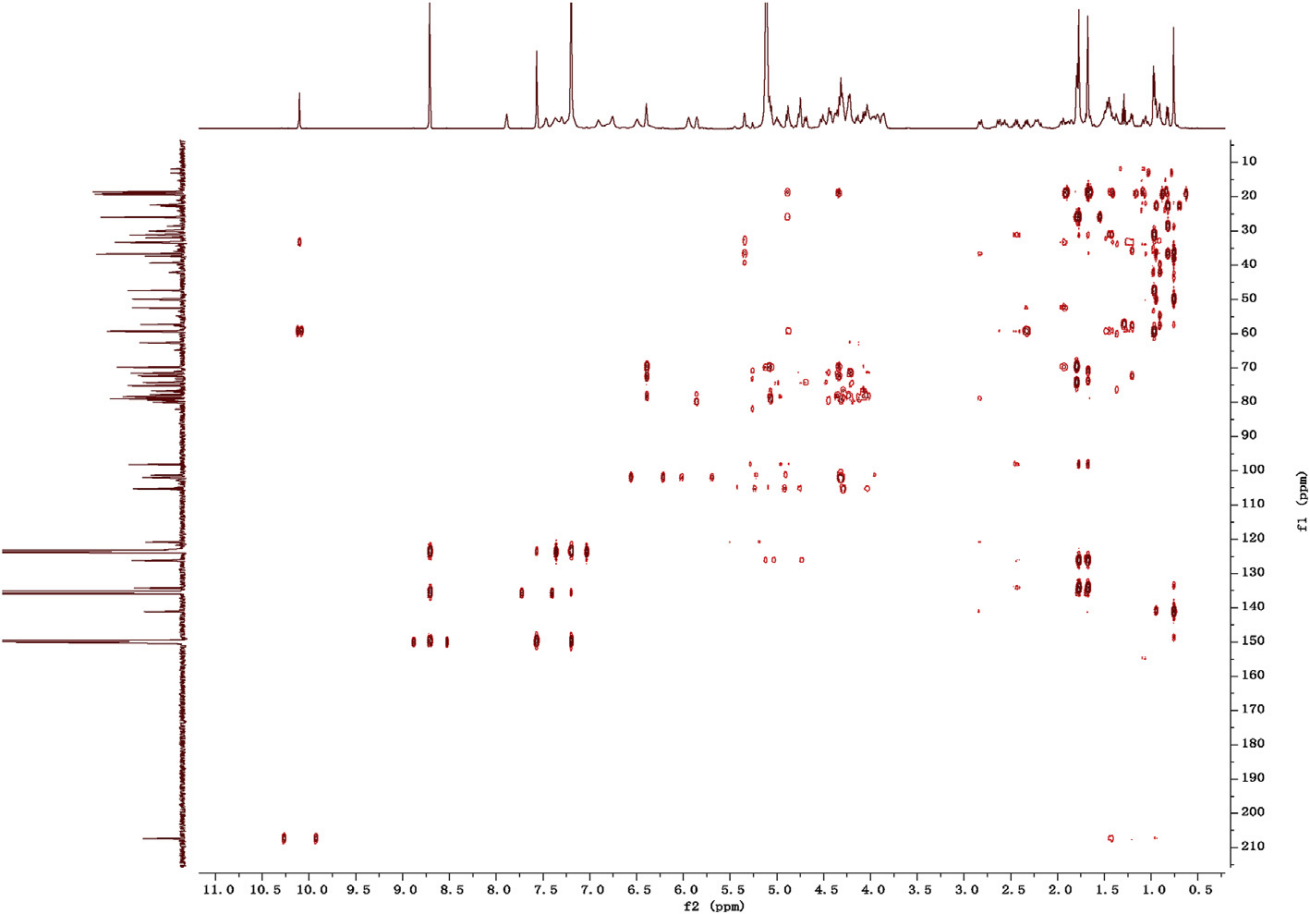
2D HMBC NMR spectrum in pyridine-*d*_5_ of 7.

**Fig. 49:**
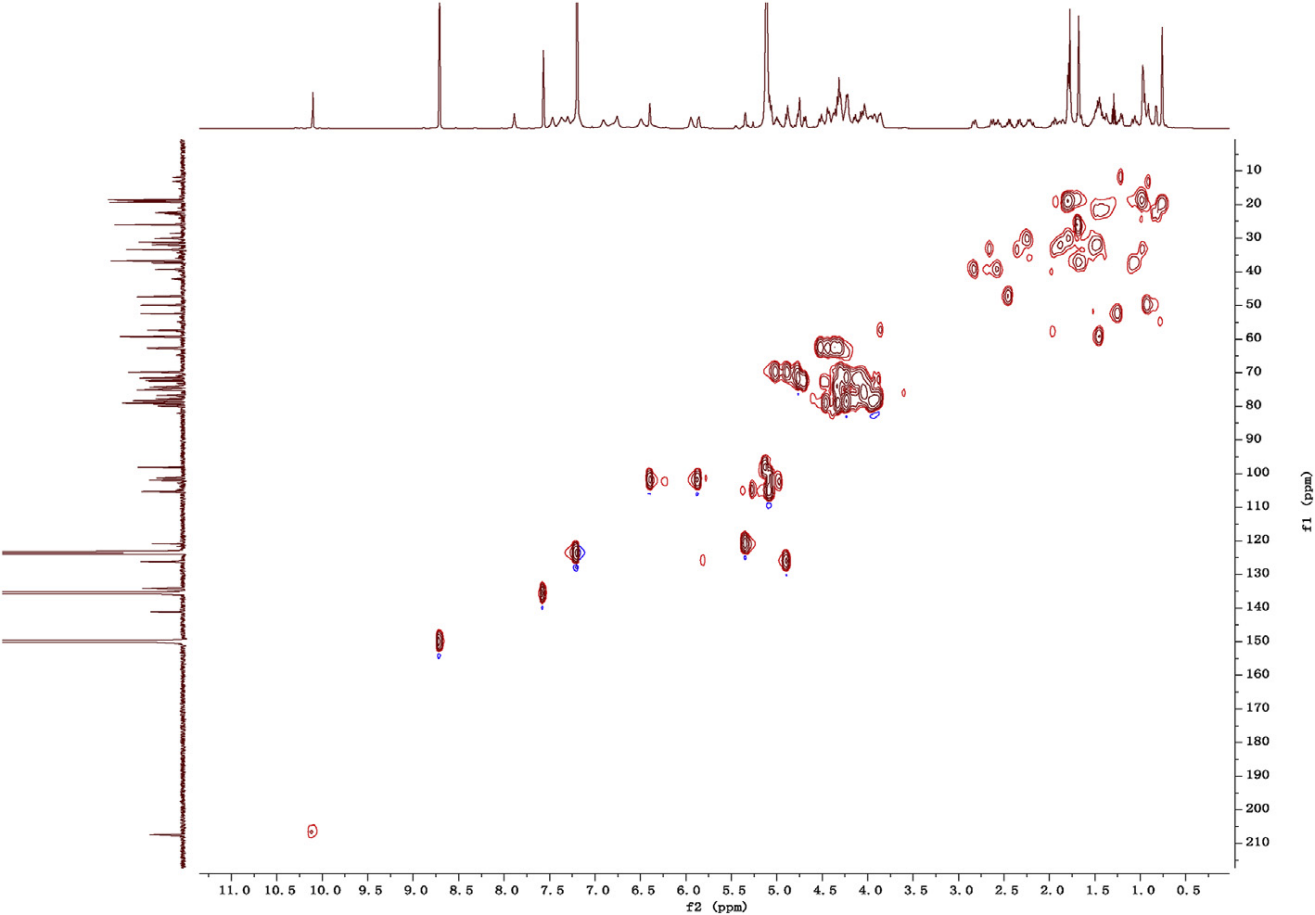
2D HSQC NMR spectrum in pyridine-*d*_5_ of 7.

**Fig. 50:**
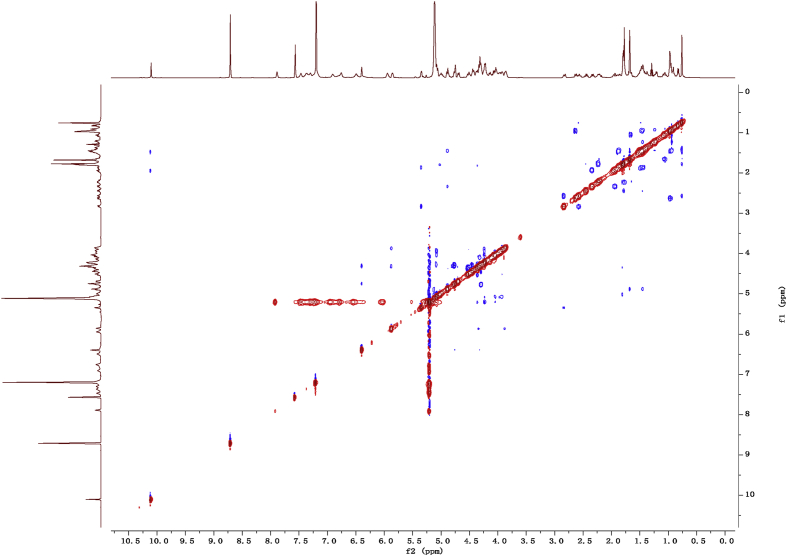
2D ROESY NMR spectrum in pyridine-*d*_5_ of 7.

**Fig. 51:**
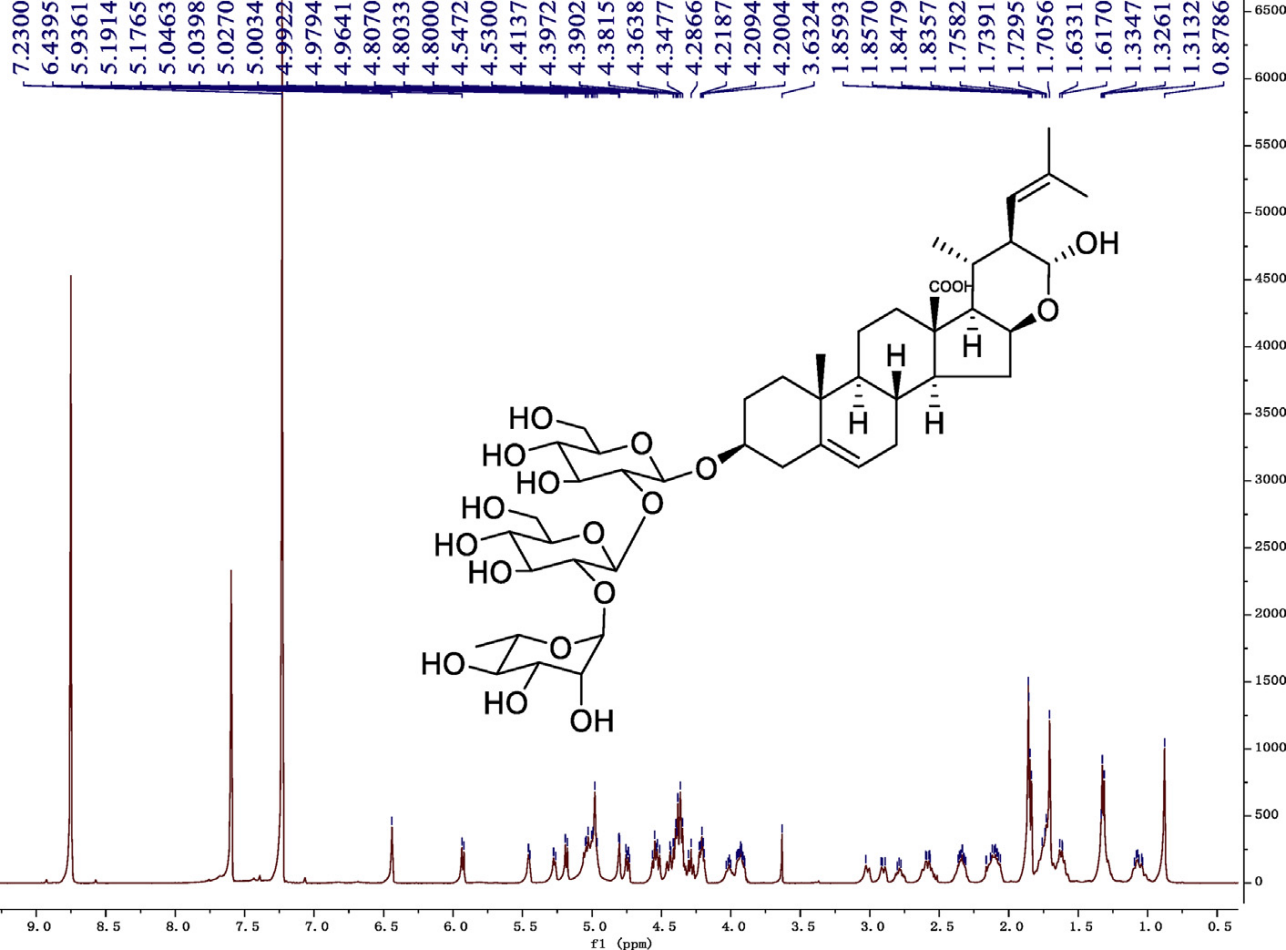
^1^H-NMR (500 MHz)spectrum in pyridine-*d*_5_ of 8.

**Fig. 52:**
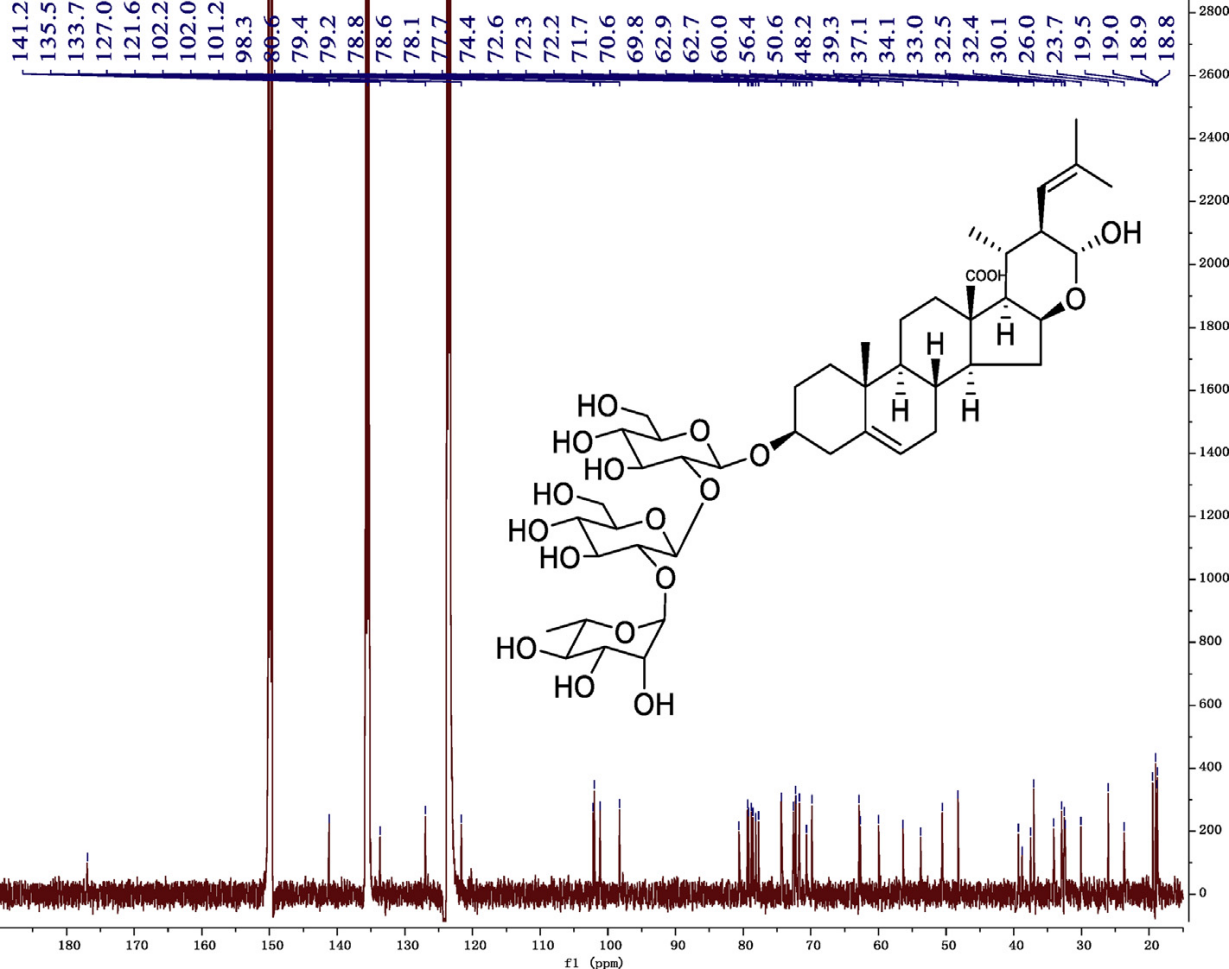
^1^C-NMR (125 MHz)spectrum in pyridine-*d*_5_ of 8.

**Fig. 53:**
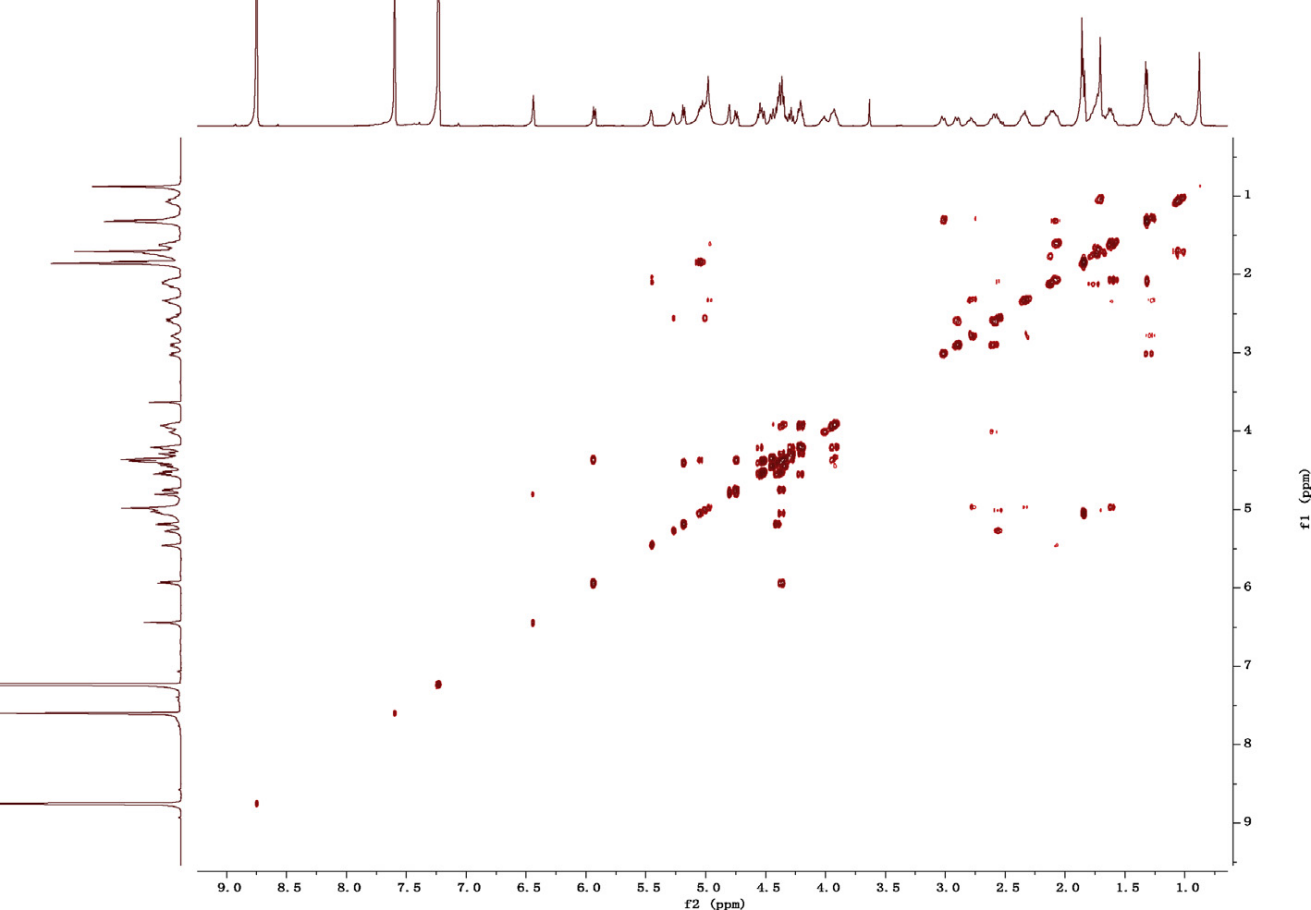
2D COSY NMR spectrum in pyridine-*d*_5_ of 8.

**Fig. 54:**
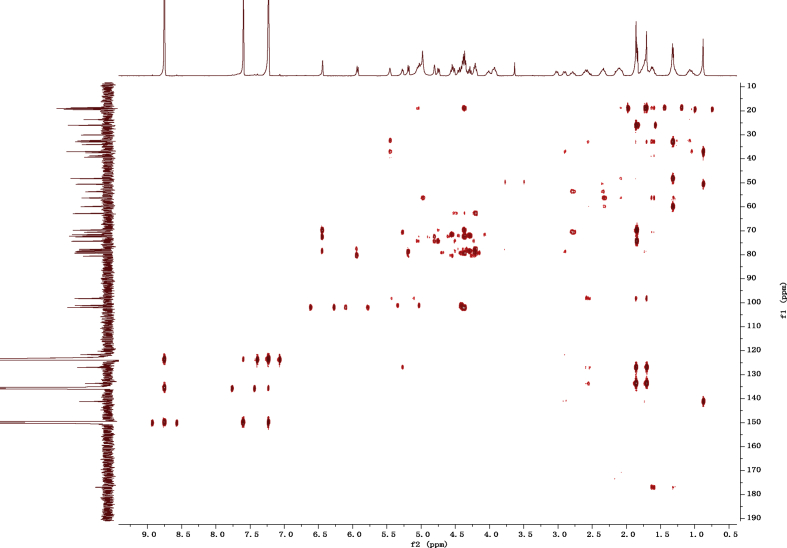
2D HMBC NMR spectrum in pyridine-*d*_5_ of 8.

**Fig. 55:**
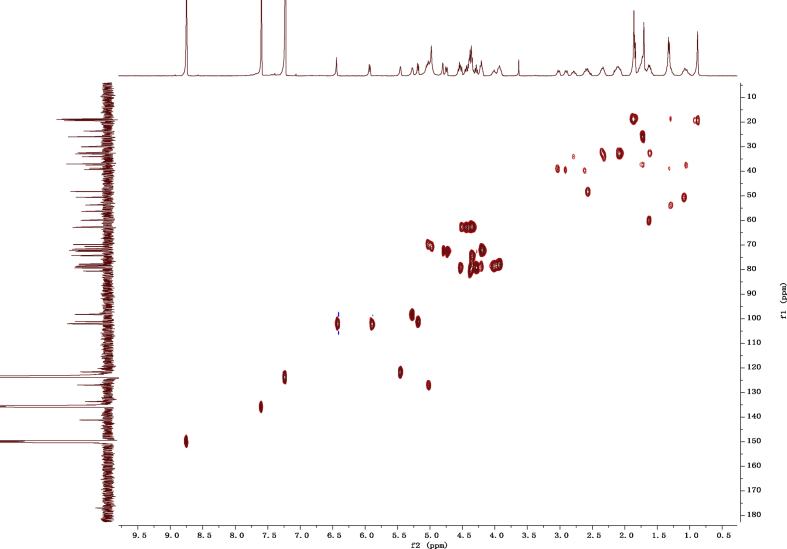
2D HSQC NMR spectrum in pyridine-*d*_5_ of 8.

**Fig. 56:**
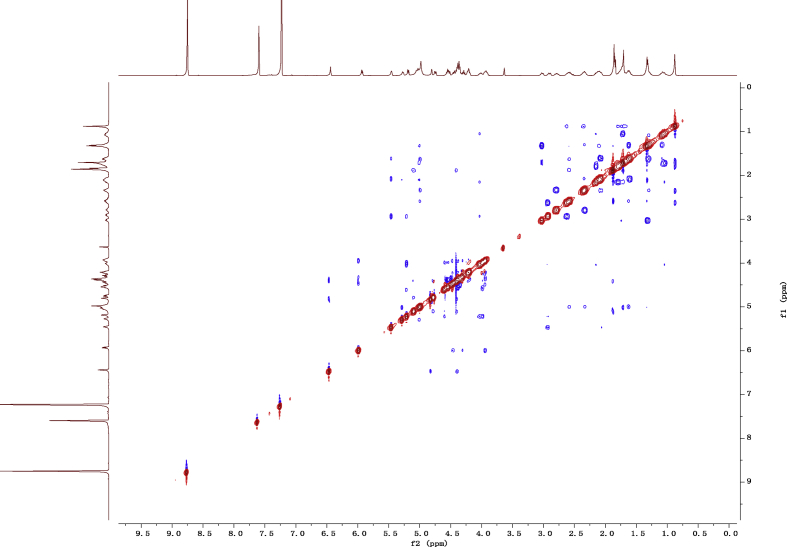
2D ROESY NMR spectrum in pyridine-*d*_5_ of 8.

**Fig. 57:**
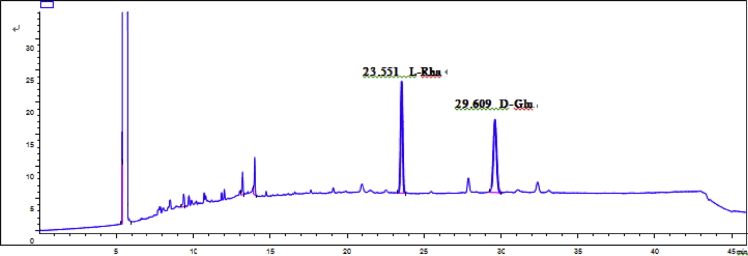
GC analysis spectra of 1.

**Fig. 58:**
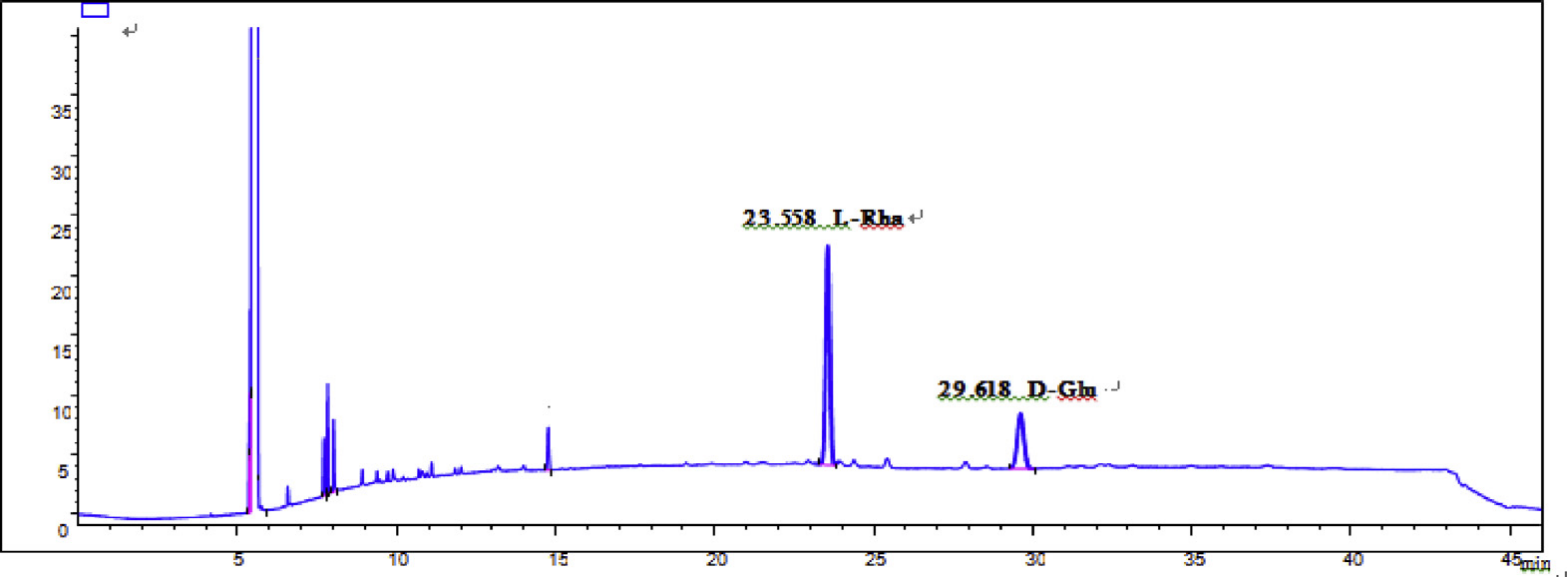
GC analysis spectra of 2.

**Fig. 59:**
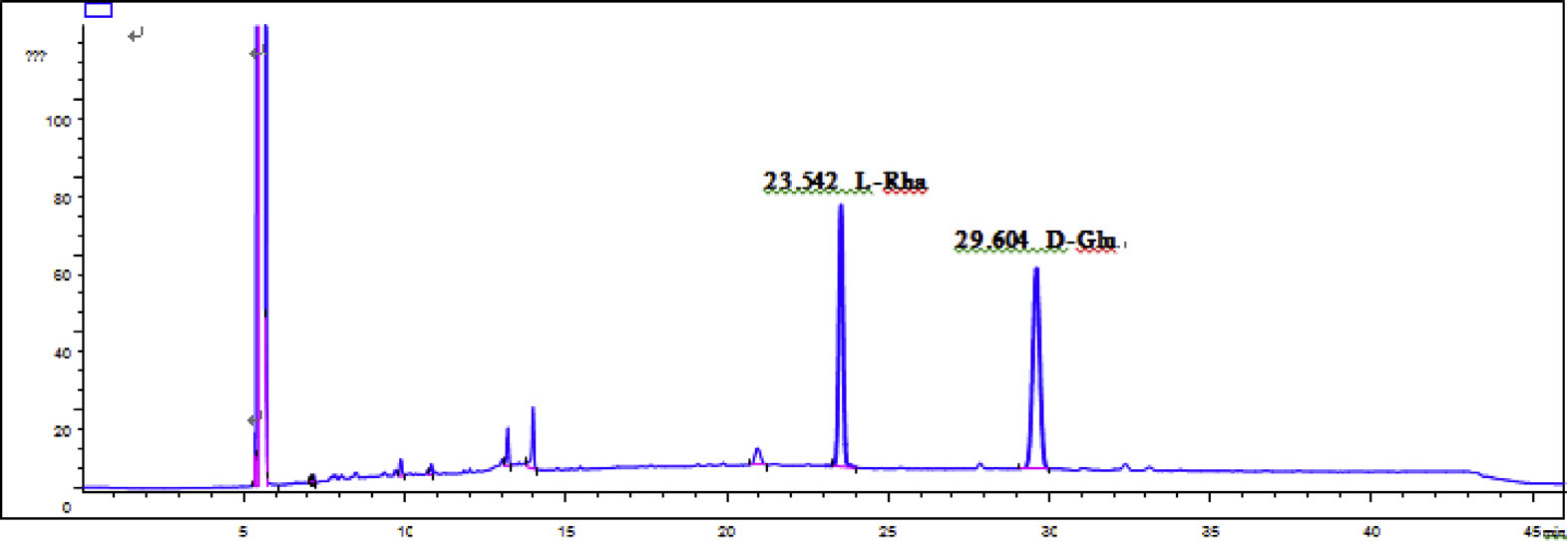
GC analysis spectra of 3.

**Fig. 60:**
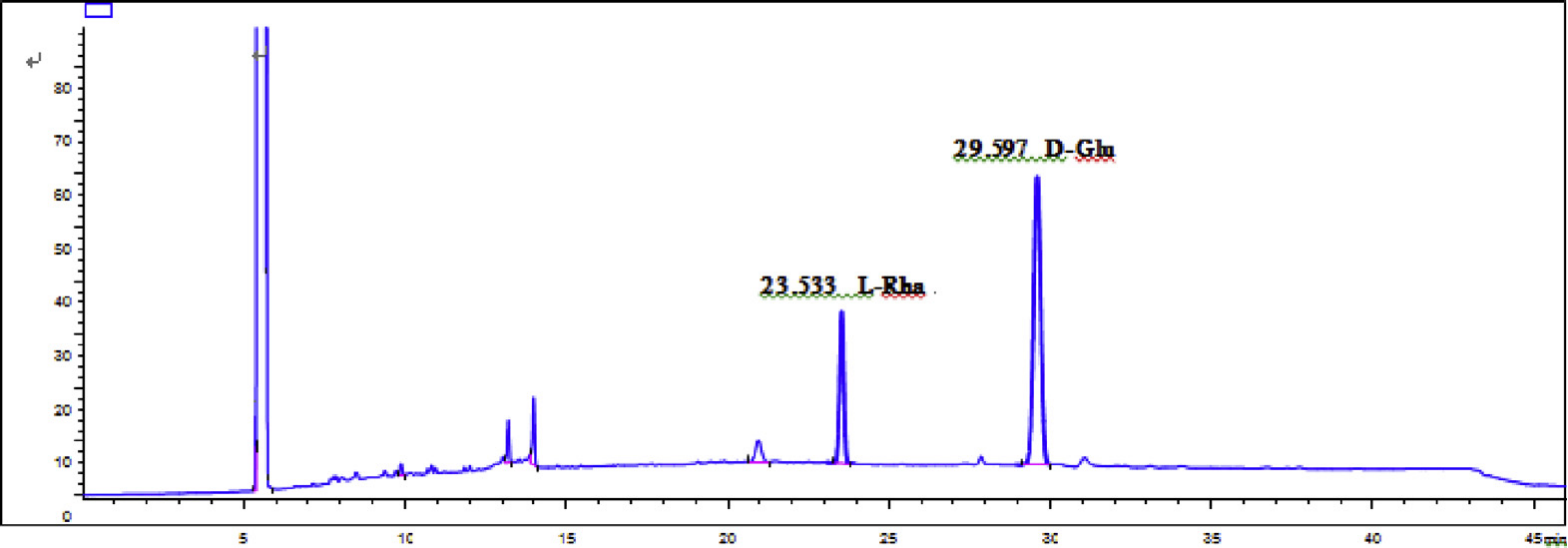
GC analysis spectra of 4.

**Fig. 61:**
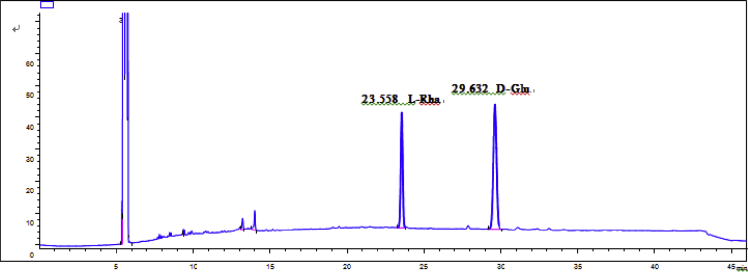
GC analysis spectra of 5.

**Fig. 62:**
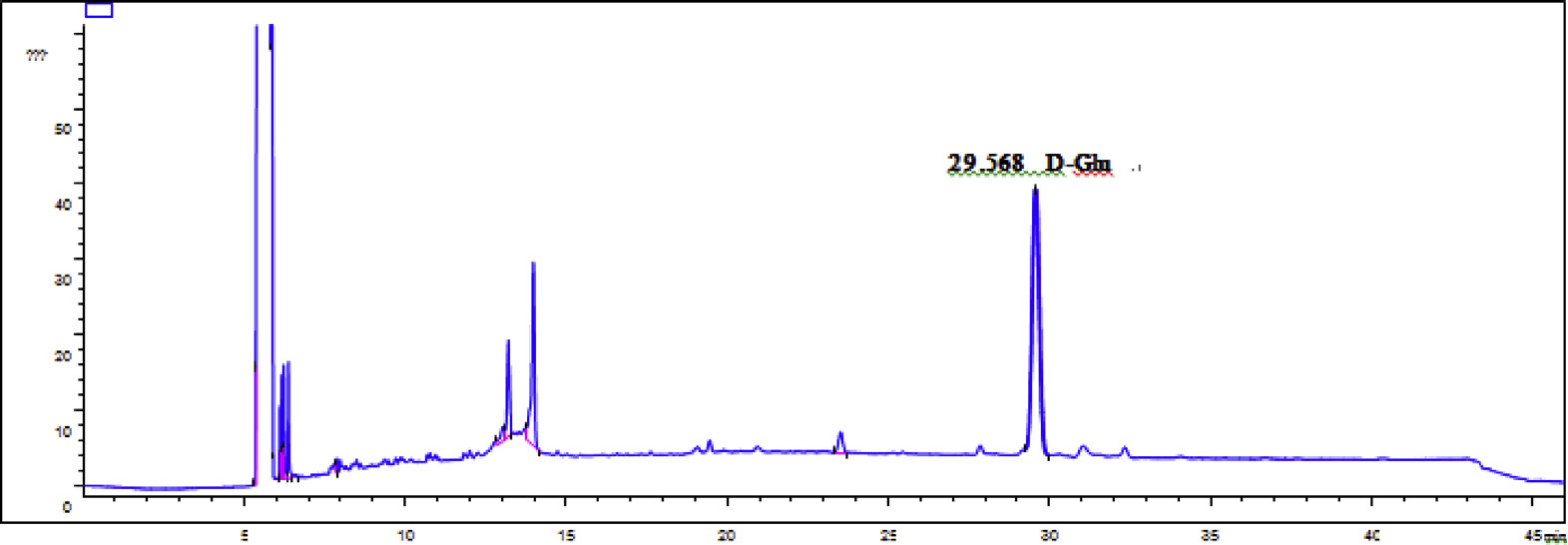
GC analysis spectra of 6.

**Fig. 63:**
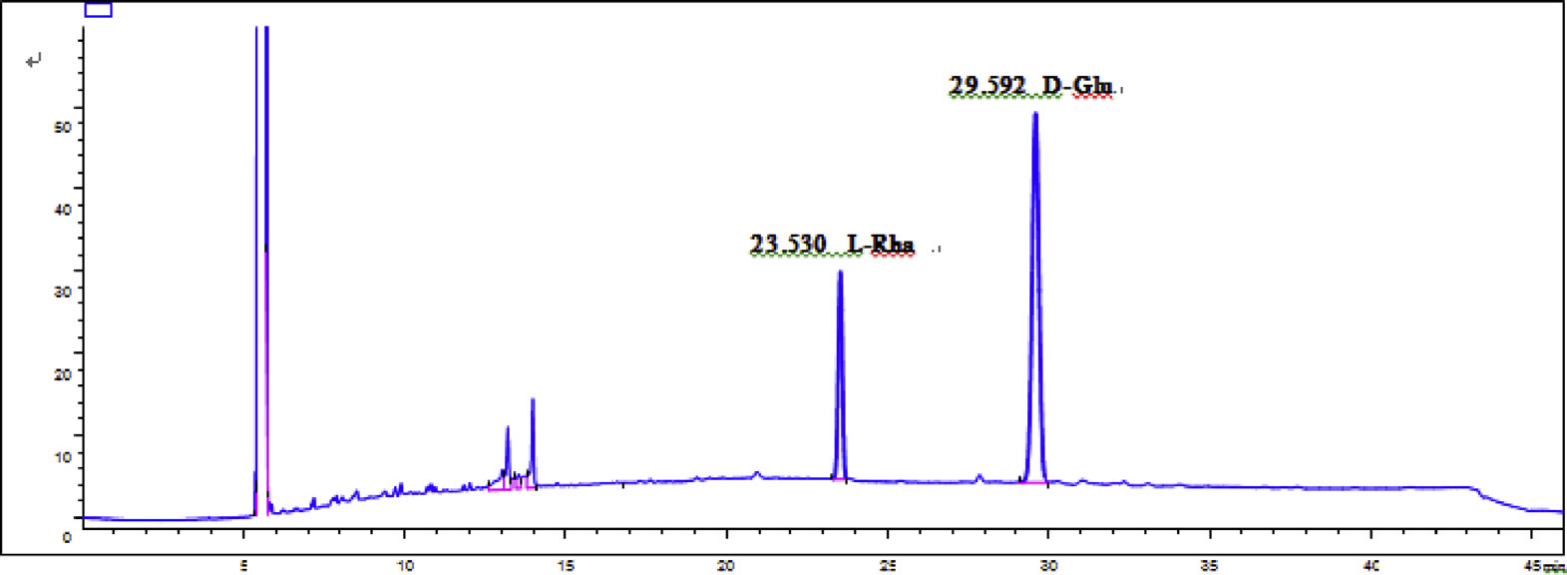
GC analysis spectra of 7.

**Fig. 64:**
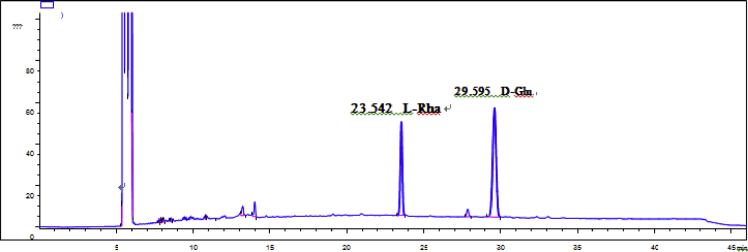
GC analysis spectra of 8.

**Fig. 65:**
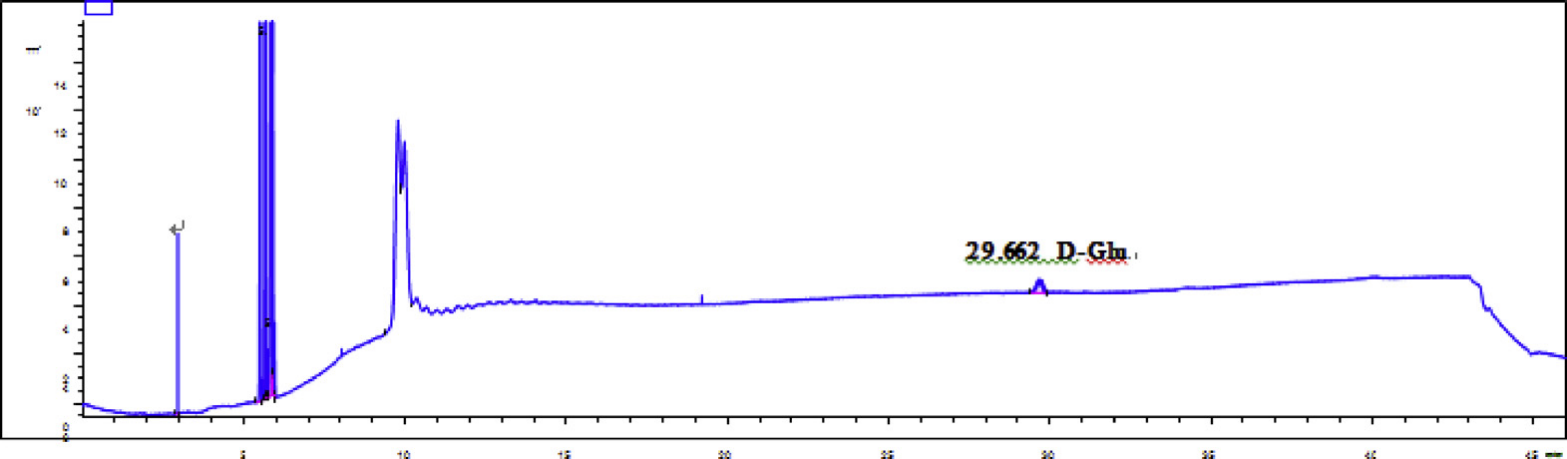
GC analysis spectra of D-Glucose.

**Fig. 66:**
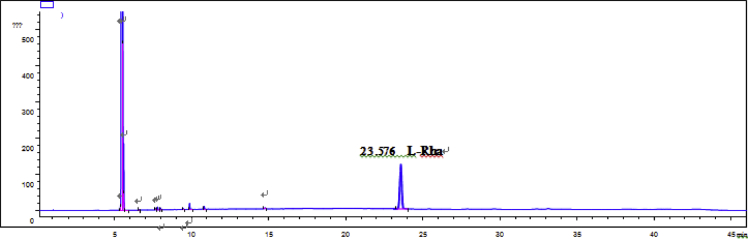
GC analysis spectra of L-rhamnose.

### 1.1 MS data for compounds 1-8

HRESIMS was performed for purified compounds 1–8. MS data was provided in Fig. 1, Fig. 2, Fig. 3, Fig. 4, Fig. 5, Fig. 6, Fig. 7, Fig. 8.

### 1.2 NMR data for compounds 1-8

1D and 2D NMR spectroscopy data of isolates 1–8 were recorded in Fig. 9, Fig. 10, Fig. 11, Fig. 12, Fig. 13, Fig. 14, Fig. 15, Fig. 16, Fig. 17, Fig. 18, Fig. 19, Fig. 20, Fig. 21, Fig. 22, Fig. 23, Fig. 24, Fig. 25, Fig. 26, Fig. 27, Fig. 28, Fig. 29, Fig. 30, Fig. 31, Fig. 32, Fig. 33, Fig. 34, Fig. 35, Fig. 36, Fig. 37, Fig. 38, Fig. 39, Fig. 40, Fig. 41, Fig. 42, Fig. 43, Fig. 44, Fig. 45, Fig. 46, Fig. 47, Fig. 48, Fig. 49, Fig. 50, Fig. 51, Fig. 52, Fig. 53, Fig. 54, Fig. 55, Fig. 56. These data include ^1^H-NMR, ^13^C-NMR, ^1^H-^1^H correlated spectroscopy (COSY), ^1^H-^13^C heteronuclear multiple bond correlation (HMBC), heteronuclear singular quantum correlation (HSQC) and rotating-frame overhauser effect spectroscopy (ROESY) (Fig. 9, Fig. 10, Fig. 11, Fig. 12, Fig. 13, Fig. 14, Fig. 15, Fig. 16, Fig. 17, Fig. 18, Fig. 19, Fig. 20, Fig. 21, Fig. 22, Fig. 23, Fig. 24, Fig. 25, Fig. 26, Fig. 27, Fig. 28, Fig. 29, Fig. 30, Fig. 31, Fig. 32, Fig. 33, Fig. 34, Fig. 35, Fig. 36, Fig. 37, Fig. 38, Fig. 39, Fig. 40, Fig. 41, Fig. 42, Fig. 43, Fig. 44, Fig. 45, Fig. 46, Fig. 47, Fig. 48, Fig. 49, Fig. 50, Fig. 51, Fig. 52, Fig. 53, Fig. 54, Fig. 55, Fig. 56).

### 1.3 GC data

Gas chromatography analysis were performed to determine the absolute configuration of sugar moieties in compounds 1–8 (Fig. 57, Fig. 58, Fig. 59, Fig. 60, Fig. 61, Fig. 62, Fig. 63, Fig. 64, Fig. 65, Fig. 66).

## 2 Experimental design, materials and methods

### 2.1 HRESIMS analysis

After isolation and purification from the bulbs of *O. saundersiae* Baker, eight cholestane glycosides were subjected to HRESIMS analysis, which was performed using an Agilent 6520 HPLC-Q-TOF (Agilent Technologies, Waldbronn, Germany).

### 2.2 NMR analysis

Cholestane glycosides were dissolved in C5D5N, respectively. Next, NMR spectra of these glycosides were acquired using a Bruker AV-Ⅲ-500 spectrometer or a Bruker-600 NMR spectrometer for ^1^H-NMR (500MHz or 600MHz) or for ^13^C-NMR (125MHz or 150MHz) at 25 °C.

### 2.3 GC analysis

The absolute configuration determination of sugar moieties was achieved by GC analysis in Agilent 7890 system equipped with a flame ionization detector (FID) for analysis. A non-polar HP-5 (60 m × 0.25 mm, with a 0.25 μm film) capillary column was applied to separate compounds.

## Conflict of interests

The authors declare that they have no known competing financial interests or personal relationships that could have appeared to influence the work reported in this paper.
